# Tuning the Performance of Metalloporphyrin-Based Pressure-Sensitive Paints through the Nature of the Central Metal Ion

**DOI:** 10.1021/acsomega.4c09045

**Published:** 2024-12-18

**Authors:** Elliott J. Nunn, Dimitrios Tsioumanis, George F. S. Whitehead, Tom B. Fisher, David A. Roberts, Mark K. Quinn, Louise S. Natrajan

**Affiliations:** † Department of Chemistry, University of Manchester, Oxford Road, Manchester M13 9PL, U.K.; ‡ Department of Mechanical and Aerospace Engineering, University of Manchester, Oxford Road, Manchester M13 9PL, U.K.; § BAE Systems, Warton Aerodrome, Warton PR4 1AX, U.K.; ∥ Aircraft Research Association, Manton Lane, Bedford MK41 7PF, U.K.

## Abstract

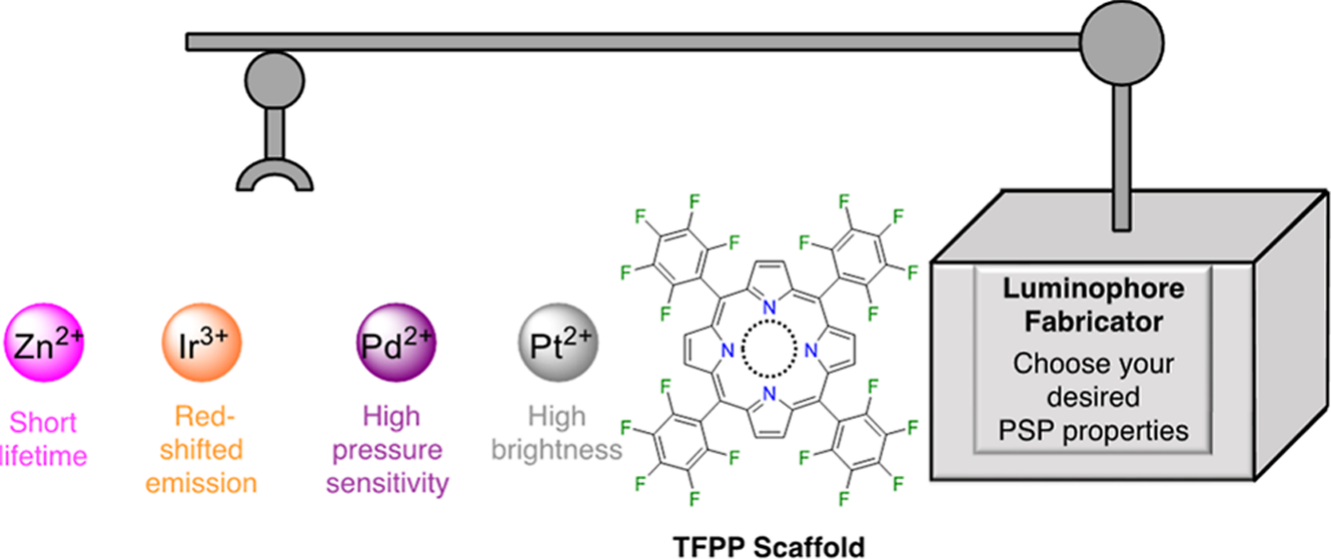

Since the 1980s, pressure-sensitive paint (PSP) has been used as an optical pressure sensor for measuring surface pressure on aircraft models in wind tunnels. Typically, PSPs have utilized platinum(II)-5,10,15,20-tetrakis(2,3,4,5,6-pentafluorophenyl)-porphyrin due to its high pressure sensitivity, phosphorescence lifetime of ∼50 μs, reasonable quantum yield of emission, and resistance to photo-oxidation. This work investigates the photophysics and electronic structure of metal complexes of 5,10,15,20-tetrakis(2,3,4,5,6-pentafluorophenyl)-porphyrin, namely, Zn(II), Pd(II), and Ir(III), as potentially improved luminophores for polymer-based PSPs. The metal ion was found to preferentially stabilize the a_2u_ MO of the porphyrin with increasing electronegativity, thus blue-shifting absorption/emission maxima and increasing Q-band intensity. The lifetime and quantum yield of emission increased and decreased, respectively, in the order of Pt(II) to Ir(III) to Pd(II), primarily due to the heavy atom effect. The increase in phosphorescence lifetimes resulted in the pressure sensitivity of the PSPs increasing in the order of Pt(II) to Ir(III) to Pd(II). However, the temperature sensitivity at pressures >70 kPa also increased with increasing phosphorescence lifetime. Overall, this work identified that the central metal ion of porphyrin luminophores can be used to tailor the resulting lifetime of the luminophore and therefore heavily influences the pressure and temperature sensitivity of polymer PSP formulations. This new insight into luminophore design can be used to optimize PSPs for a desired application.

## Introduction

Wind tunnel tests of aerospace vehicles and aircraft are increasingly making use of pressure-sensitive paint (PSP). PSP is an optical oxygen sensing technique that involves the formulation of an oxygen-sensitive phosphorescent compound, known as a luminophore, and an oxygen-permeable binder into a paint, which is then applied to the surface of a desired test model.^1^ When this coated model is placed into a wind tunnel, the static air pressure distribution causes different levels of oxygen diffusion into the binder at different locations. If light of an appropriate wavelength illuminates the model, the luminophore is excited into a singlet excited state and, through intersystem crossing, can be converted to a long-lived triplet excited state (T_1_). From the T_1_ excited state, the luminophore can relax back to the ground state via release of a lower energy photon, through a process called phosphorescence. The phosphorescent intensity is dependent on the local oxygen concentration and therefore, can be imaged and related to the surface pressure through in situ or a priori calibration.^1^ The PSP method offers advantages over traditional aerodynamic measurements, such as pressure taps, because of its global resolution and nonintrusive application. Work over the past 30 years has demonstrated the efficacy of PSP in accurately studying various aerodynamic applications such as steady and unsteady flows,^2−9^ rotating flows,^10−12^ cryogenic experiments,^13−15^ and even blast waves.^16,17^

One of the main drawbacks concerning PSP measurements is the inherent temperature sensitivity, resulting in inaccurate pressure determination. This results from nonradiative decay of the luminophore T_1_ state and temperature-dependent oxygen diffusion through the paint binder.^1^ Attempts to resolve the temperature sensitivity issue have involved the development of dual-luminophore PSPs that contain a pressure-sensitive luminophore and a secondary temperature-sensitive but pressure-insensitive luminophore. The secondary signal can then be used to account for errors in temperature change associated with the primary luminophore signal.^5,18,19^ However, some dual-luminophore formulations suffer from luminophore cross-talk which can hinder PSP performance.^20,21^

**Figure 1:**
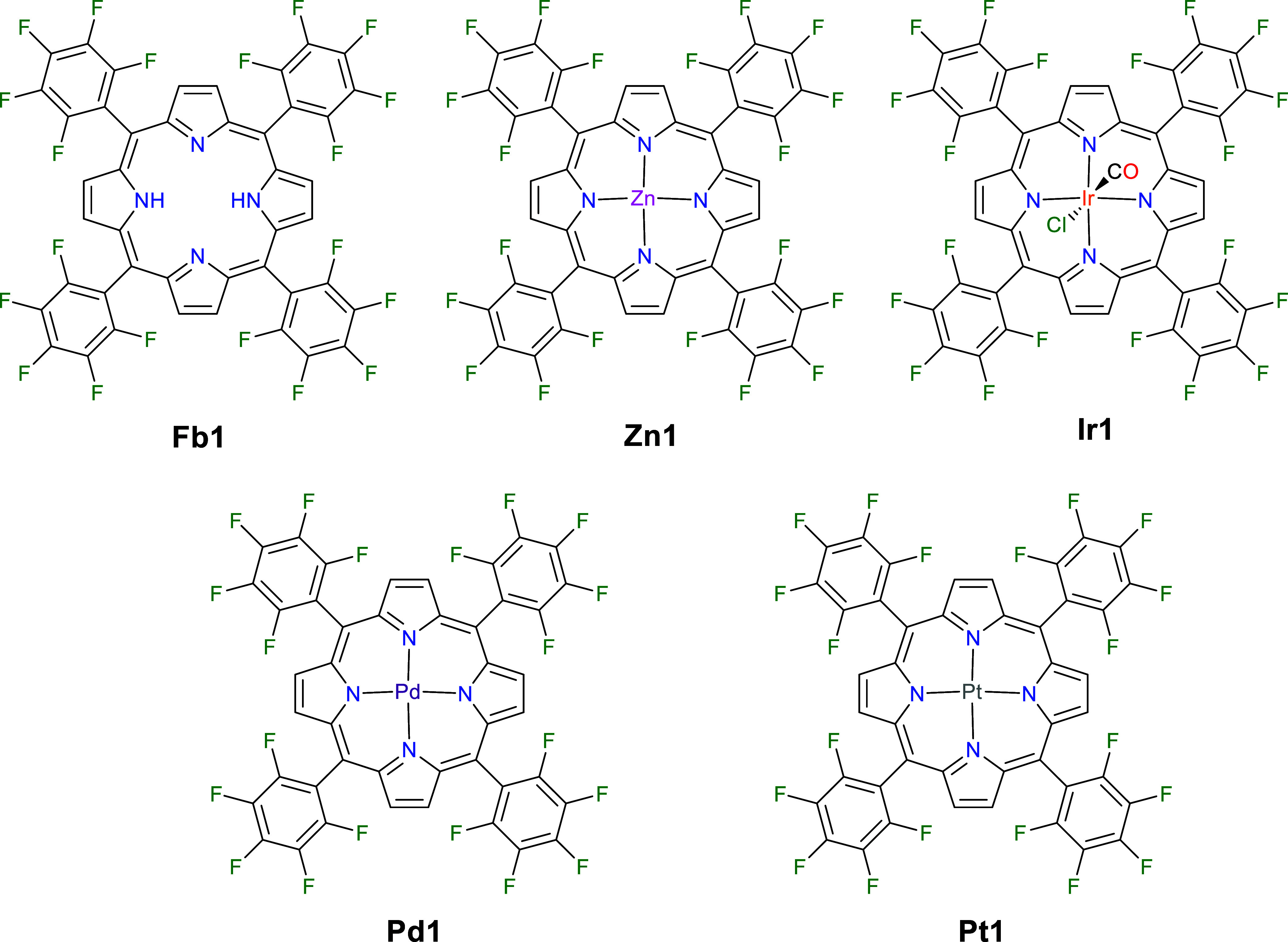
Chemical structures of the freebase porphyrin **Fb1** and the metalloporphyrins **Zn1**, **Ir1**, **Pd1**, and **Pt1**.

PSP development in recent years has largely focused on binder development, such as new polymers,^22−26^ poly ceramic PSP (PC-PSP),^27,28^ and anodized aluminum PSP (AA-PSP),^29−31^ in an attempt to improve response times for unsteady flow measurements. However, a distinct lack of development of the luminophore species has been pursued. Luminophore molecular design can be used to tailor the lifetime/quantum yield of emission and the molar absorptivity of the resulting PSP, thus affecting properties like pressure sensitivity S_p_, temperature sensitivity, S_T_, and the brightness of emission. Additionally, the solubility of a luminophore, and its interaction with the binder matrix, can greatly impact an optical molecular-based sensor’s performance.^32^ Traditionally, PSP formulations have used Pt(II) porphyrins, Ru(II) polypyridyls, and pyrene derivatives as the luminophore.^1^ Ru(II) polypyridyls have high quantum yields and adsorb well onto porous binders.^33^ However, Pt(II) porphyrin-based PSPs typically have a higher S_p_ and a relatively lower S_T_ but do not adsorb well onto porous binders. For traditional polymer-based PSPs, the luminophore Pt(II)-5,10,15,20-tetrakis(2,3,4,5,6-pentafluorphenyl)-porphyrin (PtTFPP) is most ubiquitously used due to its high degree of photostability and solubility in many common PSP polymers.^34^ Indeed, the only commercially available PSPs utilize PtTFPP as the luminophore.^35^ We recently explored the effect of tetraphenyl porphyrin degree, type, and position of phenyl halogenation on PSP performance and found these factors can have a large effect on the subsequent polymer-based PSP performance.^36^ This was especially true with S_T_, finding that PtTFPP polystyrene PSPs possessed the highest S_T_. Through luminophore design, we demonstrated that S_T_ can be reduced by 0.5%/K. However, the S_p_ in comparison was largely unaffected by porphyrin phenyl halogenation due to their similar lifetimes in the polymer binder matrix. Considering these results, this study investigates the effect of the central metal ion on the photophysics of freebase and metal complexes of TFPP and the resulting effect on polymer-based PSP performance. Typical phosphorescent metalloporphyrins constitute Pt(II), Pd(II), and Ir(III). Therefore, we introduce Ir(III)(Cl)(CO)TFPP (**Ir1**) for the first time in PSP formulations along with PtTFPP (**Pt1**) and PdTFPP (**Pd1**) to explore the effect of the central metal ion on the PSP performance (Figure 1). Additionally, Zn(II)TFPP (**Zn1**) and the freebase porphyrin, TFPP (**Fb1**), were included to study fluorescent porphyrins in polymer-based PSPs.

## Experimental Section

Detailed synthetic procedures and characterization data can be found in the accompanying Supporting Information.

### Photophysical Studies

UV–vis electronic absorption spectra were recorded on a Mettler Toledo UV5Bio spectrophotometer. Steady-state emission and excitation spectra and lifetime data were recorded on an Edinburgh Instruments FP920 Phosphorescence Lifetime Spectrometer equipped with a 450 W steady state xenon lamp, a 5 W microsecond pulsed xenon flash lamp (with single 300 mm focal length excitation and emission monochromators in Czerny Turner configuration), interchangeable EPL pulsed diode lasers, and a red-sensitive photomultiplier in Peltier (air cooled) 53 housing (Hamamatsu R928P). Plotting, fitting, and analysis of data were carried out using Origin 2019b. All data were fitted with exponential decay models and the goodness of fit evaluated by residual, χ^2^, and R^2^ analysis. These measurements were recorded in chloroform which was dried over 4 Å molecular sieves and degassed using three freeze–pump–thaw cycles and standard Schlenk techniques. All samples were prepared in an Innovative Technologies System Two, under argon, where the concentration of oxygen and water was always kept below 0.1 ppm. Emission spectra and lifetime measurements were recorded in J. Young’s taps sealed quartz cuvettes. Quantum yields were calculated using the relative method, with tetraphenyl porphyrin in toluene (Φ = 0.07) as a standard. Polystyrene-doped samples were prepared by drop casting approximately 100 μL of the PSP polystyrene solution in chloroform onto a glass microscope slide and air-dried. These samples were subsequently taken into an argon-filled glovebox to remove all residual volatiles and sealed with another glass slide using vacuum grease around the edges to prevent diffusion of oxygen into the sample. The reported data are an average of three independent measurements. The weight-averaged emission lifetimes in argon-saturated polystyrene, ⟨τ⟩ were calculated using eq 1.
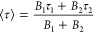
1where *B* is the initial intensity of a given component and τ is the lifetime of a given component.

### PSP Recipes

The polystyrene-based PSPs were formulated by dissolving 1 g of polystyrene (Sigma-Aldrich *M*_w_ ∼ 380,000) in 25 mL of chloroform and using a luminophore loading of 0.68% wt/wt luminophore/polystyrene.

### PSP Performance Studies

PSP formulations were sprayed onto Ambersil matt white RAL 9010 base-coated aluminum coupons using a spray gun in 12 light coats. The samples were left to air-dry for 30 minutes after spraying.

The performance of the PSP formulations was investigated in the standard approach of a priori calibration using the University of Manchester PSP calibration chamber. The a priori calibration was performed using the published procedure.^36^

The pressure sensitivity at a certain temperature, *S*_P_(*T*) was calculated from the slope of the modified Stern–Volmer calibration plots using 2 with *I*_ref_ and *P*_ref_ as the luminescence intensity and pressure, respectively, at 100 kPa and 293 K.
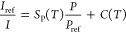
2

The temperature sensitivity at a given pressure, *S*_T_(*P*), is calculated as the percentage change in *I*_ref_/*I* with respect to the temperature by using 2 with *I*_ref_ as the luminescent intensity at 100 kPa and 293 K.
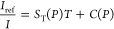
3

Photodegradation studies were conducted at room temperature and pressure and involved recording images of the PSP luminescence every 3 min for 45 min of constant illumination. The amount of photodegradation was calculated as the % luminescent intensity (at 45 min) of the original luminescent intensity (at 0 min).

## Results and Discussion

### Synthesis

The freebase porphyrin **Fb1** was synthesized via the standard Lindsey conditions in a 19% yield.^37^ The Zn(II) complex (**Zn1**) was synthesized by heating **Fb1** with Zn(OAc)_2_ to reflux in a chloroform–methanol mixture, giving an isolated yield of 86%. The Ir(III) complex (**Ir1**) was synthesized by heating **Fb1** with Ir(COD)_2_Cl_2_ to reflux in 1,2,4-trichlorobenzne, affording an isolated yield of 46%.^38^ The Pd(II) and Pt(II) (**Pd1** and **Pt1**) complexes were synthesized by heating PdCl_2_/PtCl_2_ to reflux in benzonitrile under argon, before adding **Fb1**, resulting in isolated yields of 92% and 87%, respectively (see the accompanying Supporting Information for full synthetic procedure and characterization data).^36^

### Single-Crystal X-ray Crystallography

**Figure 2:**
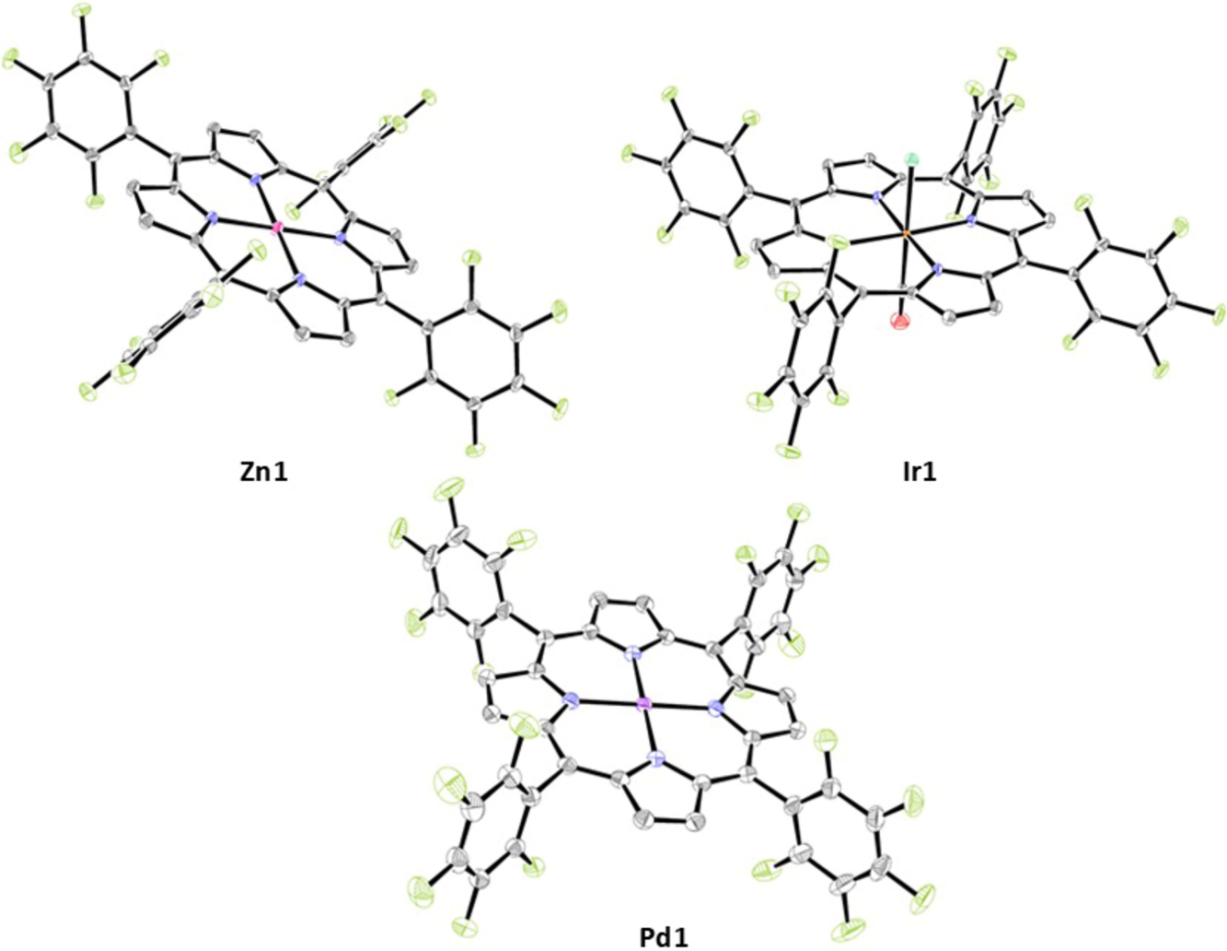
Solid-state structures of (top) **Zn1** and **Ir1** (bottom) **Pd1**. Thermal ellipsoid probability set at 50% with hydrogens omitted for clarity. Colors are assigned: C = gray, N = blue, O = red, F = light green, Cl = dark green, Zn = pink, Ir = orange, Pd = purple.

Suitable crystals for single-crystal XRD were grown through slow evaporation of a 1:1 DCM/hexane mixture for **Zn1**, **Pd1** and 1:1 DCM/chloroform for **Ir1**. The single-crystal XRD structure for **Pt1** has been previously published (CCD deposition number: 2338068).^36^ Normal coordinate structural decomposition (NSD) was used to evaluate the in-plane (IP) and out-of-plane (OOP) distortions adopted by the different porphyrins in their single-crystal geometries. NSD analysis was performed using the web-based program developed by Kingsbury and Senge.^39^ The structures of the metalloporphyrins (Figure 2) are all relatively flat, with **Pt1**, **Pd1**, and **Ir1** all having a small Δoop = 0.03 Å and the metal ion sitting in the plane of the 4 nitrogen atoms (Table 1). **Pt1** and **Pd1** adopt a slight wave (*E*_gx_/*E*_gy_), while **Ir1** adopts a slight dome (A_2u_) conformational distortion. **Zn1** has a much larger Δoop = 0.15 Å than the other metalloporphyrins, adopting a wave (*E*_gx_/*E*_gy_) conformational distortion. The porphyrins also possess IP distortions, which are expansions of the porphyrin core around the central metal ion. **Zn1** has the highest Δip = 0.19 Å and a Zn–N bond distance of 2.044(14) Å, which is consistent with other Zn(II) porphyrins, Zn–N(range) = 2.298(7) – 1.799(8) Å.^40^ Zn(II) has low-energy 3d orbitals, which are unable to π-backbond with the high-energy porphyrin π* orbitals; therefore, a weak and long Zn–N bond is formed. Looking at **Pd1**, the Δip = 0.08 Å and the Pd–N bond distance = 2.017(16) Å, which is consistent with other Pd(II) porphyrins, Pd–N(range) = 2.131(4) – 1.926(12) Å.^40^ The Pd(II) ion forms a stronger M–N interaction because of the higher in energy 4d orbitals, and thus greater π-backbonding can occur. **Pt1** has a Δip = 0.07 Å and a Pt–N bond distance of 2.009(2) Å, which is consistent with other Pt(II) porphyrins, Pt–N(range) = 2.069(4) – 1.960(10) Å.^40^ For the Pt(II) porphyrin, the M–N interaction is even stronger because the Pt(II) ion has even higher energy 5d orbitals, which facilitates even stronger π-backbonding. The Ir–N bond in **Ir1** is markedly longer at 2.050(3) Å.

**Table 1:** Key NSD and Structural Parameters of the Solid-State Structures of the Metalloporphyrins

porphyrin	out-of-plane deformation (Å)						distortion parameters (Å)		structural parameters (Å)
	B_2u_	B_1u_	A_2u_	E_g(x)_	E_g(y)_	A_1(u)_	Δoop	Δip	M–N bond distance
**Zn1**	0	0	0	0.14	0.06	0	0.15	0.19	2.044(14)
**Ir1**	0	0	0.02	0	0	0.02	0.03	0.18	2.050(3)
**Pd1**	0	0	0	0.03	0.02	0	0.03	0.08	2.017(16)
**Pt1**^a^	0	0	0	0.01	0.02	0	0.03	0.07	2.009(2)

a Data previously published.^36^

### UV–Vis Electronic Absorption Spectroscopy

**Figure 3:**
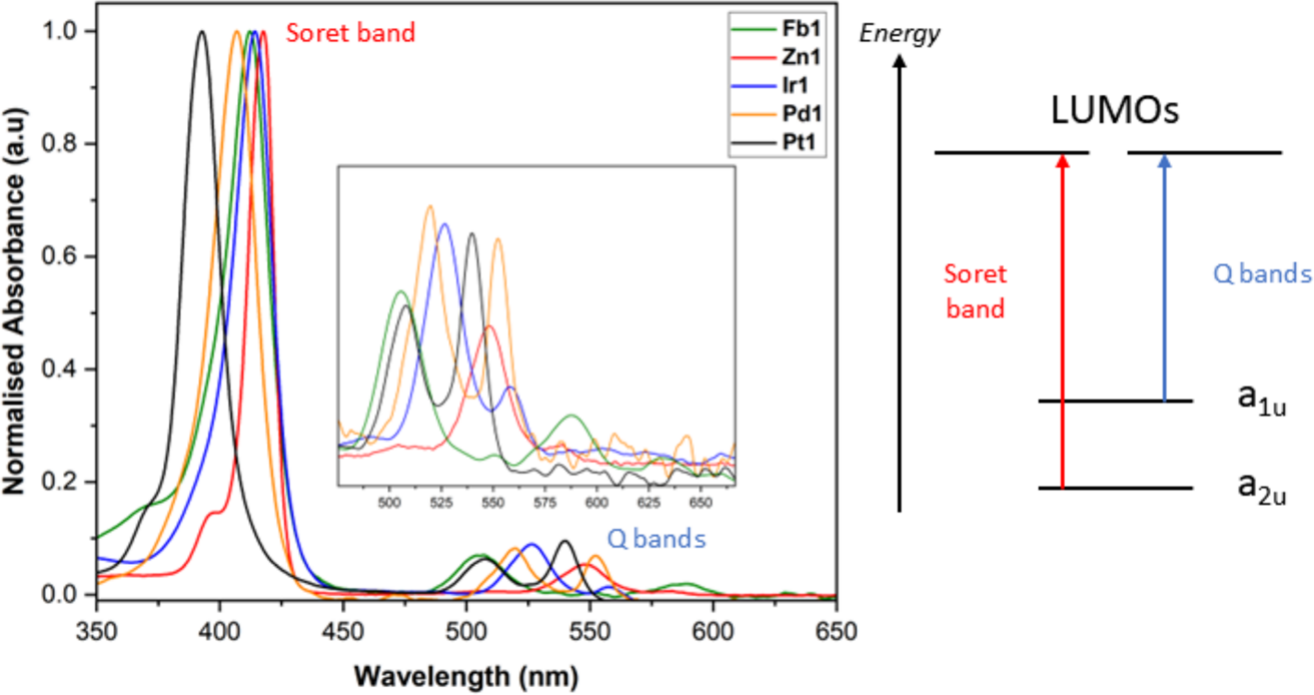
UV–vis absorption spectra of **Fb1**, **Zn1**, **Ir1**, **Pd1**, and **Pt1** in chloroform at a concentration of 2 μM. All values are normalized to the Soret band maximum. Inset shows the zoomed-in Q-band region of 500 to 675 nm. A simplified energy-level diagram showing the four Frontier orbitals of the porphyrins, and the principal electronic transitions that form the Soret and Q bands is also provided.

The UV–vis electronic absorption spectra of all metalloporphyrins in chloroform were found to be characteristic of typical metalloporphyrins, with an intense Soret band (B band) around 400 nm and two much weaker in intensity Q bands centered on 550 nm (Figure 3 and Table 2).

**Table 2:** Soret and Q Band Maxima with Molar Absorption Coefficients, ε in M^–1^ cm^–1^ in Parentheses, of the Porphyrins in Chloroform^a^

porphyrin	Soret band maximum (nm) [ε M^–1^ cm^–1^]	Q-band maxima (nm) [ε M^–1^ cm^–1^]				integrated intensity of Q(0,0)/Q(1,0)
		Q1	Q2	Q3	Q4	
**Fb1**	412 [314,900]	507 [22,300]	537 [2820]	583 [7100]	637 [1200]	0.12
**Zn1**	414 [468,600]	524 [22,200]	578 [3000]			0.25
**Ir1**	414 [176,800]	527 [19,100]	558 [6000 ]			0.34
**Pd1**	407 [271,400]	519 [22,200]	552 [18,800]			0.53
**Pt1**^b^	392 [296,500]	507 [18,800]	540 [28,400]			0.79

a The ratio of the integrated intensity of Q_abs_(0,0) to the integrated intensity of Q_abs_(1,0) is also presented (for **Fb1**, this is calculated as [Q_*y*_(0,0) + Q_*x*_(0,0)/Q_*y*_(1,0) + Q_*x*_(1,0)]).

b Data previously published.^36^

The spectral features can be explained using Gouterman’s four orbital model, where the four Frontier orbitals: highest occupied molecular orbital (HOMO)–1 (a_1u_), HOMO (a_2u_), and two degenerate lowest unoccupied molecular orbitals (LUMOs) (e_g_) are relatively isolated in energy.^41^ The Soret band is formed predominantly of a π–π* transition from HOMO–1 to the degenerate LUMOs. Whereas the Q bands are formed of Q_abs_(1,0) and Q_abs_(0,0), which are assigned as a vibronic satellite and the electronic origin π–π* transitions from the HOMO to the degenerate LUMOs. Freebase porphyrins (Fb) are known to have a further split in each Q-band due to the symmetry break of the central proton axis. Consequently, for **Fb1**, there are two sets of Q bands, Q_*y*_ and Q_*x*_, along with their accompanying vibrational satellites, resulting in four Q bands in total. The position of the Soret band blue-shifts in the order of **Ir1** = 414 nm and **Zn1** = 414 nm > **Fb1** = 412 nm > **Pd1** = 407 nm > **Pt1** = 392 nm. The reason for this blueshift has recently been revisited with Ghosh et al. finding that the metal ions stabilize the a_2u_ HOMO–1 increasing the HOMO–1 to LUMOs energy gap and thus the Soret band energy.^42^ The intensity of the Q_abs_(0,0) band increases across the family of porphyrins. In contrast, the intensity of the vibronic satellite, Q_abs_(1,0), remains roughly consistent because it derives its absorption strength from the B transitions (that make up the Soret band) via Herzberg–Teller coupling, which exceeds the intensity from the normal Franck–Condon progressions.^43^ The increasing intensity of Q_abs_(0,0) can best be seen using the ratio of the integrated intensity of Q_abs_(0,0) to Q_abs_(1,0) band (because the intensity of the Q(1,0) band remains roughly constant), which increases in the order of **Fb1** = 0.12 < **Zn1** = 0.25 < **Ir1** = 0.34 < **Pd1** = 0.53 < **Pt1** = 0.79. For the heavily fluorinated porphyrin (TFPP) used in this study, the a_2u_ MO is already greatly stabilized and so the freebase porphyrin, **Fb1**, has its a_2u_ MO and a_1u_ MO flipped. Therefore, the a_2u_ becomes the HOMO–1 and the a_1u_ the HOMO.^44^ As metal ions are incorporated into this porphyrin, the a_2u_ HOMO–1 will be increasingly stabilized, with the strength of this stabilization dependent on the electronegativity of the metal. Therefore, any metal ion will stabilize the a_2u_ HOMO–1 away from the a_1u_ HOMO, decreasing orbital mixing and increasing Q_abs_(0,0) intensity. The exception to this trend is **Ir1**, which has a reduced intensity Q_abs_(0,0) band when compared to **Pd1**, despite Pd(II) and Ir(III) having similar electronegativity values (2.70 and 2.79, respectively).^45^ The axial Cl and CO act as electron-withdrawing substituents, which can lower the energy of the a_1u_ HOMO, thus bringing it closer to the a_2u_ HOMO–1, therefore, increasing the level of orbital mixing, which in turn reduces Q_abs_(0,0) intensity.

### Density Functional Theory and Time-Dependent-Density Functional Theory Calculations

To further explore the electronic structure of **Fb1**, **Zn1**, **Ir1**, **Pd1**, and **Pt1**, DFT and time-dependent DFT (TD-DFT) calculations were carried out. Calculations employed either the B3LYP^46^ (DFT) or CAM-B3LYP^47^ (TD-DFT) functional with the lanl2dz^48^ basis set for all atoms. Solvation in chloroform was treated with a conductor-like polarizable continuum model and the D4 charge-dependent atom-pairwise dispersion correction^49^ was included throughout. This methodology has been found to be optimal in previous porphyrin DFT and TD-DFT calculations.^50^ All structures are optimized and confirmed as minima on the potential energy surface, through the absence of imaginary vibrational modes. All calculations were performed by using the Orca 5.0.4 software package.

### DFT Calculations

**Figure 4:**
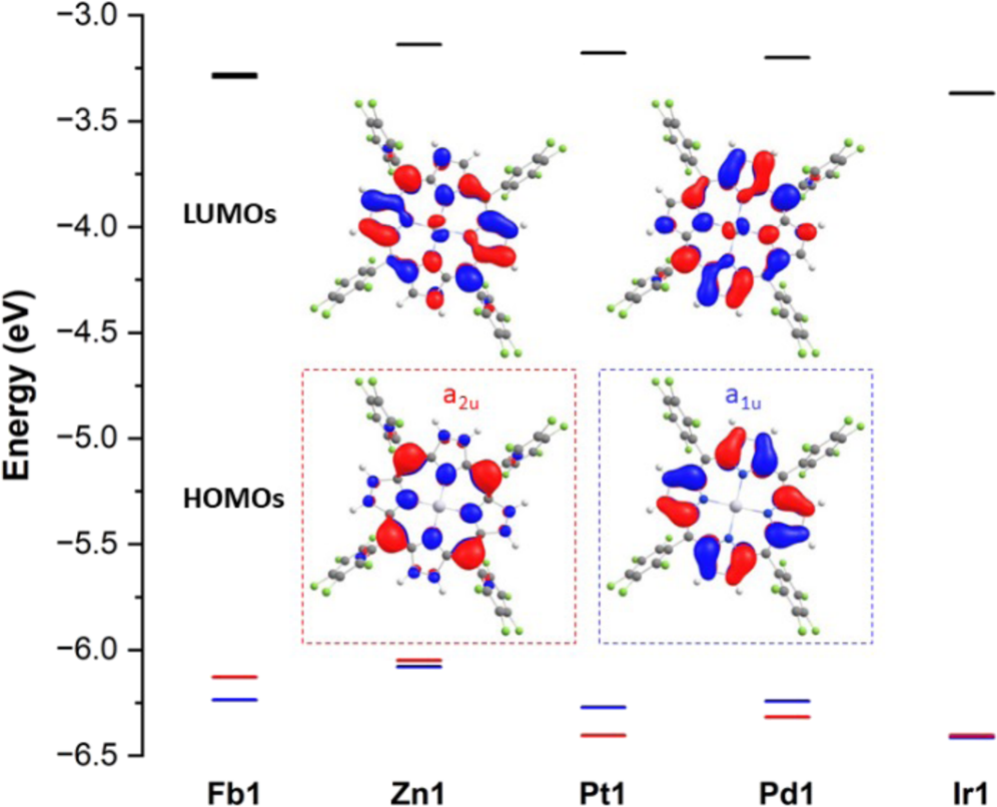
Energy-level diagram showing the energy, in eV, of the HOMOs (bottom): a_1u_ (blue) and a_2u_ (red) and the LUMOs (top) of **Fb1**, **Zn1**, **Pt1**, **Pd1**, and **Ir1**. Orbitals are drawn with an isosurface value of 0.03 e/A^3^.

All calculated structures are near-flat. The relative energies of HOMO–1, HOMO, LUMO, and LUMO+1, with the accompanying relevant energy gaps, are found in Figure 4 and Table S1. The porphyrins possess an energetically isolated degenerate LUMO and LUMO+1 (except **Fb1,** which has the LUMOs split in energy by 0.01 eV, due to the central proton axis breaking symmetry) and a close in energy HOMO and HOMO–1. The HOMO and HOMO–1 are a_2u_ and a_1u_ π MO and are identical across all of the systems studied.

Comparing **Fb1** to **Zn1**, the incorporation of the Zn(II) ion raises the HOMO and HOMO–1 in energy, and they also become closer in energy, with HOMO to HOMO–1 gaps of 0.11 and 0.03 eV, respectively. For **Pt1**, **Pd1**, and **Ir1**, the DFT predicts that the a_2u_ MO is increasingly stabilized by the increasingly electronegative metal ions, thus causing the a_2u_ MO to become the HOMO–1 as it is stabilized below the a_1u_ MO in **Pd1** and **Pt1**. Therefore, the HOMO to HOMO–1 energy gap increases in the order of **Ir1** = 0.01 eV < **Pd1** = 0.08 eV < **Pt1** = 0.13 eV. The a_2u_ MO of **Pt1** and **Ir1** are predicted to be at similar energies (−6.40 and −6.41 eV, respectively), owing to the relatively similar electronegativities of the metal ions (2.98 and 2.79, respectively). However, for **Ir1** the a_1u_ MO is greatly stabilized and the a_2u_ MO is slightly stabilized. This makes the two MOs almost degenerate in energy, contributing to the much smaller HOMO to HOMO–1 energy gap for **Ir1**. The average of the HOMO–1 to LUMO and HOMO to LUMO energy gaps affords the center of gravity of the resulting absorption spectrum, which then determines Soret band energies. The center of gravity of the absorption spectrum is predicted to increase steadily in the order of **Fb1** = 2.89 eV < **Zn1** = 2.93 eV < **Ir1** = 3.04 eV < **Pd1** = 3.08 eV < **Pt1** = 3.16 eV because the a_2u_ MO is being stabilized away from the a_1u_ MO. The exception for this is **Ir1**, which has a similar a_2u_ MO energy to **Pt1**, but its degenerate LUMOs are predicted to be stabilized by about 0.2 eV with respect to the other metalloporphyrins, leading to smaller HOMOs to LUMOs energy gaps.

### TD-DFT Calculations

**Figure 5:**
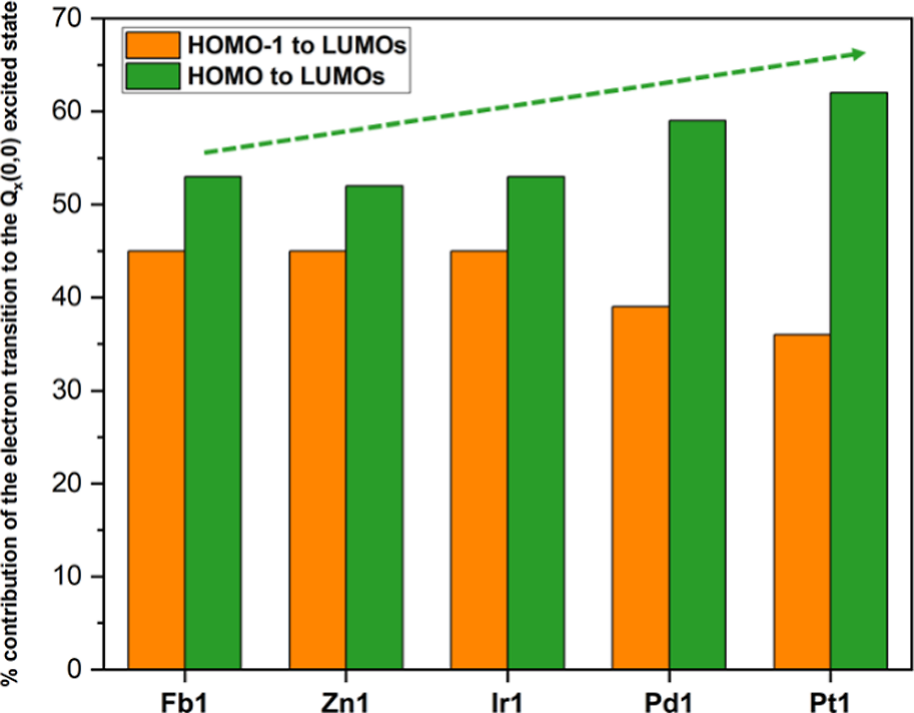
Sum of the predicted % contribution of the transitions from the HOMO–1 and HOMO to the LUMOs for the Q_*x*_ excited state from the TD-DFT calculations.

TD-DFT calculations predict the UV–vis electronic spectra generally well, with each porphyrin having four major excited states, except for **Pd1** (Table S3), in line with the four-orbital model. The absorption spectra of the metalloporphyrins are predicted to have Soret and Q bands, each made of two symmetry matched excited states, B_*y*_/B_*x*_ and Q_*y*_/Q_*x*_, respectively (with B_*y*_ and B_*x*_ forming the Soret band and Q_*y*_ and Q_*x*_ forming the Q_abs_(0,0) feature). The freebase porphyrin, **Fb1**, is predicted to have two symmetry split excited states for each absorption feature due to the central proton axis energetically splitting the LUMO and LUMO+1. Surprisingly, the Soret band feature of **Pd1** is predicted to be formed of three degenerate excited states. Across the series, Soret bands are predicted to blue-shift in wavelength, which matches the experimental data. This blueshift is due to the increasing energy gap between the LUMOs and the center of gravity of the absorption spectrum, with increasing metal ion electronegativity. However, the exception is **Ir1** with its lower in energy LUMOs, which decrease the HOMOs to LUMOs energy gap. Therefore, **Ir1** possesses a red-shifted Soret band, with respect to **Pd1** and **Pt1**. The oscillator strength of the Q-band feature is predicted to increase steadily from **Fb1** ≈ 0 < **Zn1** = 0.001 < **Ir1** = 0.003 < **Pd1** = 0.010 < **Pt9** = 0.021. This trend aligns with the experimental data, where the intensity of the Q_abs_(0,0) band increased across the series. The four orbital model predicts that each excited state is made up of a pair of one-electron transitions from the HOMO–1 and HOMO to the LUMOs.^41^ Different combinations of these one-electron transitions can happen constructively, leading to the intense Soret band feature, or destructively, leading to the much weaker Q-band features.^51^ The more mixing of the electron transitions, the more destructive combinations are possible and thus the weaker the Q bands. This orbital mixing is dependent on the energy gap between the HOMO and HOMO–1 orbitals in the ground state, which is most evident when comparing the sum of the predicted contributions of the transitions from the HOMO–1 to the LUMOs and the HOMO to the LUMOs, for a given excited state. Focusing on the Q_*x*_ excited state, for example, (Figure 5 and Table S3), the sum of the contributions of the HOMO to LUMOs transitions become increasingly more dominant over the sum of the contributions of the HOMO–1 to LUMOs transitions in the Q_*x*_ excited-state makeup across the series.

The increasing dominance of the HOMO to LUMOs transitions (and therefore, decreasing dominance of HOMO–1 to LUMOs transitions) in the Q_*x*_ excited-state makeup, follows the progression **Fb1** = 53% ≈ **Zn1** = 52% ≈ **Ir1** = 53% < **Pd1** = 59% < **Pt1** = 62%. The HOMO to LUMOs transitions become increasingly dominant in the excited-state composition from **Zn1** to **Pd1** to **Pt1** due to the increasing stabilization of the a_2u_ MO, resulting in a greater HOMO to HOMO–1 energy gap. However, for **Ir1** the a_1u_ MO is also stabilized, which decreases the HOMO to HOMO–1 energy gap. As less orbital mixing occurs in the excited state, the Q bands increase in intensity because fewer destructive combinations are possible. It is useful to look at the resulting MO energy levels in the excited structures to better explain the excited-state composition (Table S2). Upon excitation, the now singly occupied molecular orbital (SOMO)–LUMO/LUMO+1 destabilize in energy and the now SOMO–HOMO/HOMO–1 stabilize in energy. The a_2u_ MO stabilizes in energy to a greater extent than the a_1u_ MO, causing the a_2u_ MO to become the HOMO–1 in all metalloporphyrins. Whereas in **Fb1**, the a_1u_ MO remains as the HOMO–1. Consequently, the HOMO to HOMO–1 energy gaps in the excited structures are **Fb1** = 0.01 eV < **Zn1** = 0.06 eV < **Ir1** = 0.08 eV < **Pd1** = 0.16 eV < **Pt9** = 0.22 eV, which matches the experimentally observed increasing intensity of the Q_abs_(0,0) feature across this series.

### Emission Spectroscopy

**Figure 6:**
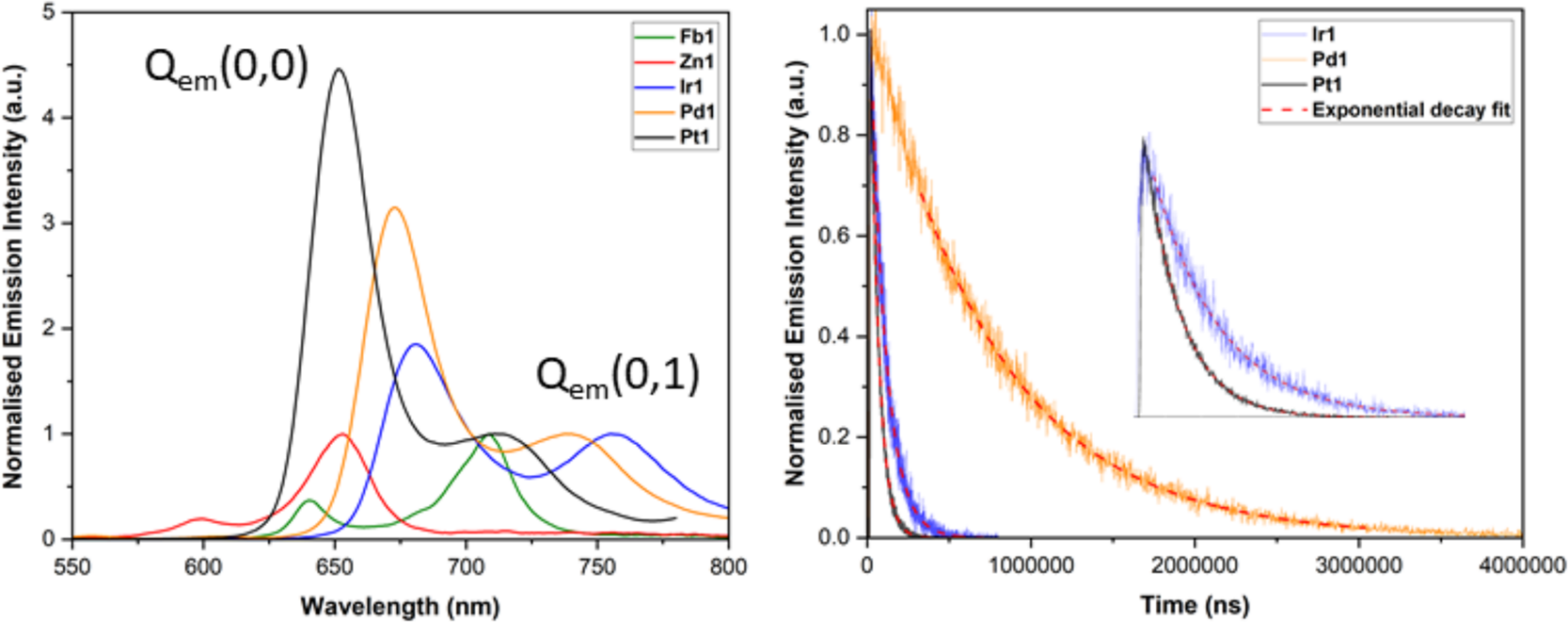
(Left) Emission spectra of **Fb1**, **Zn1**, **Ir1**, **Pd1**, and **Pt1** in deoxygenated chloroform at a concentration of 0.5 μM. All values are normalized to the Q_em_(0,1) maximum of the respective porphyrin to highlight increasing Q_em_(0,0) intensity. Excitation was in the Soret band maximum of the respective porphyrin. (Right) Lifetimes of emission for **Ir1**, **Pd1**, and **Pt1** in deoxygenated chloroform at a concentration of 0.5 μM, fitted to a monoexponential decay. Inset shows a zoomed in section to highlight the decays of **Ir1** and **Pt1**. The lifetimes and associated fits for **Fb1** and **Zn1** can be found in the accompanying Supporting Information.

The emission spectra of the porphyrins in chloroform possess 2 distinct bands, the electronic origin of the emission, denoted as Q_em_(0,0) and a lower in energy vibronic satellite, denoted as Q_em_(0,1) (Figure 6 and Table 3). The Q_em_(0,0) maxima follow the same trend as the Soret maxima with **Ir1** = 680 nm > **Pd1** = 672 nm > **Pt1** = 651 nm. However, the Q_em_(0,0) maximum of **Zn1** is blue-shifted, with respect to the other metalloporphyrins to 599 nm, which is common for Zn(II) porphyrins.^52^ Similar to the absorption spectra, the Q_em_(0,0) feature increases in intensity with increasing metal ion electronegativity, for example, the integrated intensity of Q_em_(0,0)/Q_em_(0,1), increases in the order of **Fb1** = 0.26 > **Zn1** = 0.16 < **Ir1** = 0.98 < **Pd1** = 1.30 < **Pt1** = 1.76. The increasing intensity of the spectral features suggests that the HOMO to HOMO–1 energy gap is increasing across this series.

**Table 3:** Emission Peak Maxima, Lifetimes of Emission, and Quantum Yields of Emission in Deoxygenated Chloroform^a^

porphyrin	Q_em_(0,0) maximum (nm)	Q_em_(0,1) maximum (nm)	integrated intensity of Q(0,0)/Q(0,1)	lifetime of emission (deoxygenated)τ_(Ar)_ (μs)	quantum yield of emission (deoxygenated)Φ_(Ar)_
**Fb1**	640	708	0.26	0.0096	0.046
**Zn1**	599	653	0.16	0.0013	0.013
**Ir1**	680	755	0.98	90.0	0.022
**Pd1**	672	739	1.30	748.2	0.039
**Pt1**^b^	651	710	1.76	49.6	0.082

a The ratio of the integrated intensity of Q_em_(0,0) to the integrated intensity of Q_em_(0,1) is also presented.

b Data previously published.^36^

Lifetimes, τ_(Ar)_ and quantum yields, Φ_(Ar)_, were measured in argon-saturated solutions and can be split into two categories: fluorescent and phosphorescent porphyrins. Focusing on the fluorescent porphyrins, **Fb1** and **Zn1**, their lifetimes are much shorter, 9.6 and 1.3 ns, respectively, which is typical of fast fluorescence from the S_1_ state. Incorporation of the Zn(II) ion promotes intersystem crossing from the S_1_ state to the T_1_ state through increased spin orbital coupling via the heavy atom effect. Therefore, **Zn1** has a much shorter τ_(Ar)_ and a lower Φ_(Ar)_. Moving onto the phosphorescent porphyrins, their emission lifetimes are much longer, on the order of μs, indicating slow phosphorescence from the T_1_ state. The phosphorescence τ_(Ar)_ and Φ_(Ar)_ of **Pt1** (τ_(Ar)_ = 49.6 μs and Φ_(Ar)_ = 0.082) and **Pd1** (τ_(Ar)_ = 748.2 μs and Φ_(Ar)_ = 0.039) are governed mainly by the increasing heavy atom effect of the Pt(II) compared with that of the Pd(II) ion, which decreases τ_(Ar)_ and increases Φ_(Ar)_. **Ir1** by comparison has a smaller Φ_(Ar)_ = 0.022 than both **Pt1** and **Pd1** and a τ_(Ar)_ = 90.0 μs, which is longer than **Pt1** but shorter than **Pd1**.

### Polystyrene PSP Performance Studies

The metalloporphyrins were formulated into polymer PSPs, using the polystyrene formulation from our previous luminophore comparison study.^36^ In this study, however, a higher *M*_w_ of polystyrene was used (*M*_w_ = 380,000). Polystyrene is not the optimal polymer for PSP formulations but was deemed suitable for luminophore comparison studies. The performance metrics studied are pressure sensitivity, *S*_p_, temperature sensitivity, *S*_T_, and photodegradation after constant illumination at room temperature and pressure for 45 min.

### Pressure Sensitivity, *S*_p_

**Figure 7:**
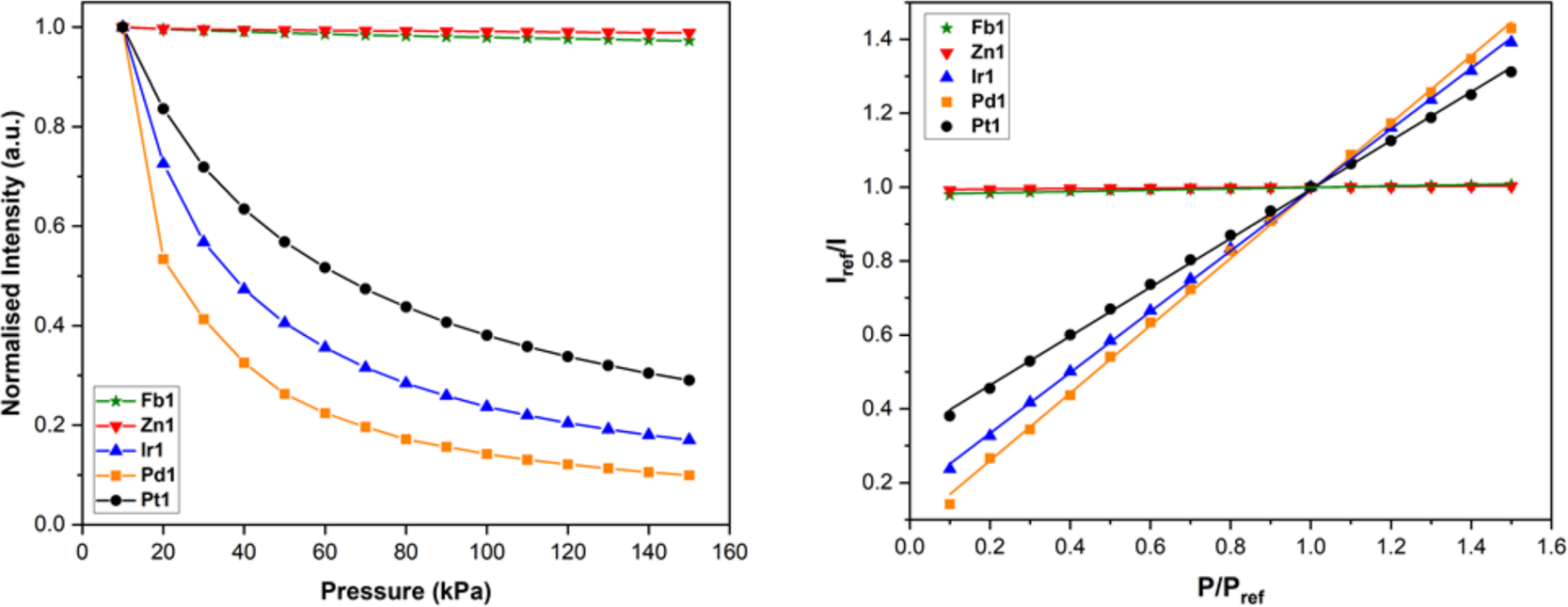
(Left) Luminescence response with increasing pressure of **Fb1**, **Zn1**, **Ir1**, **Pd1**, and **Pt1** polystyrene PSPs at 293 K. (Right) Modified Stern–Volmer calibrated luminescent response to pressure with associated linear fits of **Fb1**, **Zn1**, **Ir1**, **Pd1**, and **Pt1** polystyrene PSPs at 293 K.

Pressure sensitivity, *S*_p_, is a crucial performance metric for PSPs; a higher sensitivity to pressure allows for smaller pressure changes to be resolved. Stern–Volmer calibrated responses at 273, 293, and 313 K are presented below (Figure 7 and Table 4). *S*_p_ is calculated as the change in *I*_ref_/*I*, with respect to *P*/*P*_ref_, and is quoted at 293 K (denoted as *S*_p_ (293 K)). The phosphorescence lifetime, τ_(Ar)_, of the luminophore is an important determining factor of *S*_p_; the longer the T_1_ excited state exists, the more chances there are for collisional quenching with diffused O_2_. There is no significant change in the emission and excitation spectra of the porphyrins in polystyrene. The phosphorescent lifetimes in argon-saturated polystyrene were found to be biexponential for the phosphorescent porphyrins (this is common for luminophores immobilized in a polymer matrix) and monoexponential for the fluorescent porphyrins.^53^ For the phosphorescent porphyrins, the *S*_p_ (293 K) increases in the order of **Pt1** = 0.662 < **Ir1** = 0.824 < **Pd1** = 0.913, which correlates to the trend in the τ_(Ar)_ of these luminophores in polystyrene. Therefore, **Ir1-** and **Pd1-**containing PSPs are much more sensitive to pressure than **Pt1**-containing PSPs and are attractive options for improved PSP formulations. The small changes in *I*_ref_/*I* with increasing pressure for **Fb1** and **Zn1**, make accurate performance determination unreliable, and they would not be able to accurately sense large changes in pressure. The fluorescent porphyrins had *S*_p_ (293 K) = 0.019 for **Fb1** and 0.007 for **Zn1**. On the fluorescent time scale of nanoseconds these luminophores are very limited by oxygen diffusion through the polystyrene binder. However, the reduced fluorescent lifetime of **Zn1** may make it an attractive option for fast responding porous PSPs, where sensing is theoretically not limited by oxygen diffusion through the binder and thus is something we plan to explore in the future.^54^ Overall, the *S*_p_ (293 K) of the phosphorescent porphyrin PSPs presented here varies by 38%, highlighting that the nature of the central metal ion can have a substantial effect on *S*_p_.

**Table 4:** Pressure Sensitivity *S*_p_ of **Fb1**, **Zn1**, **Ir1**, **Pd1**, and **Pt1** Polystyrene PSPs at 273, 293, and 313 K with Associated Standard Errors^a^

porphyrin	pressure sensitivity, *S*_p_			weight-averaged emission lifetime in argon-saturated polystyrene ⟨ τ⟩ (μs)
	273 K	293 K	313 K	
**Fb1**	0.015 ± 0.001	0.019 ± 0.001	0.018 ± 0.001	0.0156
**Zn1**	–0.004 ± 0.001	0.007 ± 0.001	0.007 ± 0.001	0.00157
**Ir1**	0.564 ± 0.006	0.824 ± 0.005	1.175 ± 0.010	96.8
**Pd1**	0.624 ± 0.004	0.913 ± 0.009	1.228 ± 0.019	891.4
**Pt1**	0.448 ± 0.004	0.662 ± 0.005	0.970 ± 0.008	55.3

a These values were found from the gradient of the modified Stern–Volmer calibrated plots at each temperature. The weight average lifetimes in argon-saturated polystyrene were calculated using a bi-exponential decay fit and eq 1.

### Temperature Sensitivity, *S*_T_

**Figure 8:**
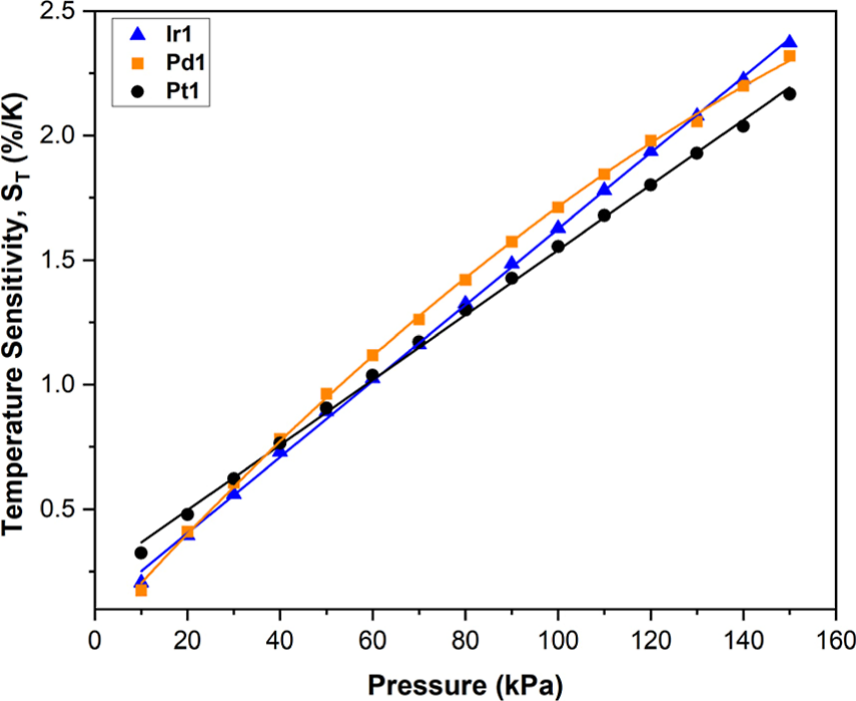
Change in temperature sensitivity, *S*_T_, of Ir1, Pd1, and Pt1 polystyrene PSPs with increasing pressure. These values were found from the change in *I*_ref_/*I* across the three temperatures of the modified Stern–Volmer calibrated plots. The linear fits (Ir1 and Pt1) and the second-order polynomial fit (Pd1) of this data are also included.

The temperature sensitivity, *S*_T_, of PSPs is also an important performance characteristic. PSPs and polymer PSPs especially possess a high inherent temperature sensitivity, which makes reliable pressure determination more convoluted. The temperature sensitivity of PSPs is due to two factors: at higher pressures the temperature-dependent oxygen diffusion through the binder dominates and at lower pressures the intrinsic temperature dependence of the nonradiative deactivation of the luminophore excited state dominates.^1^ A low *S*_T_, single luminophore PSP would be ideal to avoid the complexity associated with binary PSP formulations. The long-lived T_1_ excited state has a temperature dependency due to vibrational, rotational, and collisional quenching. Keeping the polymer binder the same, while changing the luminophore, theoretically allows investigation of the luminophore effect on PSP temperature sensitivity. S_T_ is calculated as the change in *I*_ref_/*I* with respect to the temperature and is typically quoted at 100 kPa, denoted as *S*_T_ (100 kPa). Looking at the phosphorescent porphyrins first, it can be seen that the *S*_T_ (100 kPa) increases in the following order: **Pt1** = 1.56%/K < **Ir1** = 1.62%/K < **Pd1** = 1.71%/K, aligning well with their increasing emission lifetimes. The change in S_T_ with increasing pressures is also presented for **Ir1**, **Pd1**, and **Pt1** (Figure 8). For **Ir1** and **Pd1**, *S*_T_ (10 kPa) ≈ 0.20%/K; for **Pt1**, it is slightly higher, with *S*_T_ (10 kPa) = 0.32%/K. As the pressure increases, *S*_T_ increases linearly for **Ir1** and **Pt1**. However, the increase is greater for **Ir1** than for **Pt1**, causing the *S*_T_ of **Ir1** to become greater than that of **Pt1** at roughly 65 kPa. At higher pressures, there is more oxygen in the binder matrix, and the longer lived T_1_ lifetime of **Ir1**, compared to **Pt1**, will facilitate more deactivation by this increased O_2_ concentration. Therefore, **Ir1** at these higher pressures will be more susceptible to the increasing collisions with O_2_ because of the increasing energy of diffused O_2_, which accompanies increasing temperatures. **Pd1** has an even longer T_1_ lifetime than **Ir1** and so its *S*_T_ becomes greater than that of **Pt1** earlier (roughly 40 kPa). However, in contrast to **Ir1** and **Pt1**, for **Pd1**, a second-order polynomial fit was found to better fit the change in *S*_T_ with pressure, compared to a linear fit (*R*^2^ = 0.999 for second-order polynomial and 0.991 for linear). This nonlinear behavior is probably due to excess quenching of **Pd1** at higher pressures and consequently causes the S_T_ of **Pd1** to plateau and subsequently dip below that of **Ir1** at pressures >130 kPa. These findings are interesting, as they implicate that the more pressure-sensitive **Ir1** and **Pd1** PSPs could be used in low-pressure (<70 kPa) measurements where their S_T_ is lower and their low quantum yields (at high pressures) are less of an issue. On the other hand, **Pt1**-based PSPs are more suited to high pressure (>70 kPa) applications, where the *S*_T_ is lower (compared to **Ir1** and **Pt1**) and the higher quantum yields are more desirable. The two fluorescent porphyrins, **Fb1** and **Zn1**, exhibit weak temperature sensitivities, 0.13 and 0.11%/K, respectively, due to their much shorter fluorescent lifetimes.

### Photodegradation

**Figure 9:**
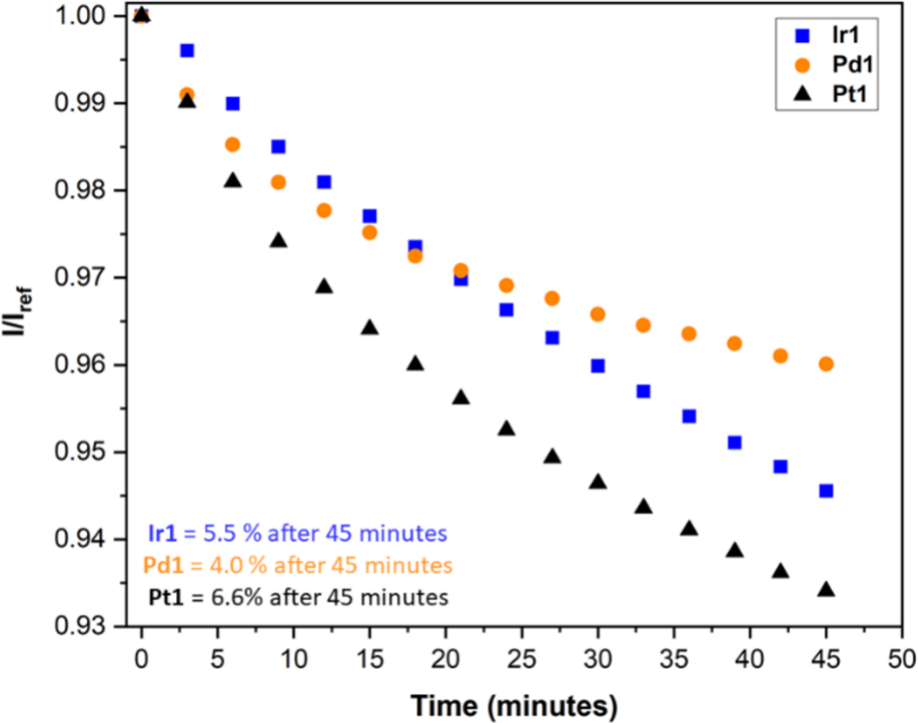
Photodegradation of **Ir1**, **Pd1**, and **Pt1** polystyrene PSPs, at room temperature and pressure, over 45 min of constant illumination. *I*_ref_ is the luminescent intensity at 0 min.

Photodegradation of the phosphorescent porphyrins **Ir1**, **Pd1**, and **Pt1** polystyrene PSPs was also investigated. The photodegradation is calculated as the % luminescent intensity of the original luminescent intensity (at 0 min) after 45 min of constant illumination at room temperature and pressure. A high-performing PSP formulation will have minimal photodegradation during testing to facilitate greater usage over multiple tests. Photodegradation of PSPs during testing occurs primarily via the formed singlet oxygen radical, which can go on to damage the luminophore and binder, leading to a reduction in paint performance.^55^**Pt1** photodegrades the most after 45 min, with a 6.6% reduction in luminescence intensity (Figure 9). **Pd1** is the most photostable, with a 4% reduction in luminescence intensity after 45 min of constant illumination. **Ir1** is more photostable than **Pt1**, with a 5.5% photodegradation after 45 min. However, the photodegradation of **Ir1** is noticeably more linear in this time scale (compared to **Pt1**), which begins to tail off toward the end of the measurement.

## Conclusions

We have investigated a family of metalloporphyrins and their parent free-base ligand TFPP to examine the effect of the central metal ion of porphyrins on the electronic structure, photophysics, and the resulting polymer-based PSP performance. The Pt(II) complex, PtTFPP (**Pt1** in this study), is used almost ubiquitously in polymer-based PSP formulations and therefore we presented Pd(II) (**Pd1**), Zn(II) (**Zn1**), and, for the first time in the PSP field, Ir(III) (**Ir1**) TFPP complexes. The central metal ion mainly affects the energy of the occupied a_2u_ MO through metal electronegativity-dependent stabilization. Preferential stabilization of the a_2u_ MO by more electronegative metals widened the HOMOs to LUMOs energy gap, resulting in a blueshift of the spectra. However, **Ir1** was an exception, as it was found using DFT calculations to have stabilized LUMOs relative to the other metalloporphyrins, resulting in red-shifted spectral features. The preferential stabilization of the a_2u_ away from the a_1u_ MO increases the HOMO to HOMO–1 energy gap and thus increases the intensity of Q(0,0) feature in the absorption and emission spectra. **Ir1** was found to have a greatly stabilized a_1u_ MO, brining it closer in energy to the a_2u_ MO, thus reducing the intensity of the Q(0,0) features. TD-DFT calculations showed that the intensity of the Q_abs_(0,0) feature increased due to the contributions of the electronic transitions from the HOMO–1 and the HOMO to the LUMOs, in the excited-state makeup decreasing and increasing, respectively, with increasing HOMO–1 to HOMO energy gaps. The heavy metal effect of the Zn(II) ion caused **Zn1** to have a reduced fluorescence lifetime and quantum yield, compared to the freebase porphyrin **Fb1**. On the other hand, **Pd1**, **Ir1**, and **Pt1** exhibited room-temperature phosphorescence, with Pt(II) having a larger quantum yield (0.039 and 0.082, respectively) but shorter lifetime of emission (748.2 and 49.6 μs, respectively) due to the increased heavy atom effect of Pt(II) over Pd(II). **Ir1** had a smaller quantum yield (0.022) than **Pd1** but a longer lifetime of emission (90.0 μs) compared to that of **Pt1**. The phosphorescent porphyrins **Ir1**, **Pd1**, and **Pt1**, were all viable candidates as luminophores in PSP formulations. The pressure sensitivity at 293 K, *S*_p_ (293 K), of the resulting polystyrene PSPs greatly increased in the order of **Pt1** = 0.662 < **Ir1** = 0.824 < **Pd1** = 0.913, in line with the increasing phosphorescence lifetimes. Increasing the phosphorescence lifetime of the luminophore was found to increase the temperature sensitivity (*S*_T_) at higher pressures, with **Ir1** and **Pd1** having higher S_T_ at higher pressures than **Pt1**. However, at lower pressures, **Pt1** had the highest *S*_T_ and **Ir1**/**Pd1** had a lower *S*_T_. Therefore, the nature of the central metal ion in porphyrin luminophores can be used to tailor PSP formulations for specific pressure measurement. The quantum yields of emission for **Pd1** and **Ir1** are also much lower than for **Pt1**, reducing the brightness of these PSPs. Finally, this work shows that the central metal ion of porphyrin luminophores can be used to tune the pressure and temperature sensitivity of the resulting PSP formulation for the desired application. This work contrasts our previous study on the effect of porphyrin phenyl halogenation, which mainly affected the resulting temperature sensitivity of the PSPs.^36^ We hope this research can assist with making new and improved PSP formulations.

## Supporting Information Available

Details and spectra of syntheses and characterization of compounds and their NMR, mass spectrometry and IR data, crystallographic data, DFT calculations, luminescence data, and polystyrene PSP performance data (PDF)
Crystallographic data for Zn(II) TFPP complex (CIF)
Crystallographic data for Ir(III) TFPP complex (CIF)
Crystallographic data for Pd(II) TFPP complex (CIF)

## Supplementary Material

ao4c09045_si_001.pdf

ao4c09045_si_002.cif

ao4c09045_si_003.cif

ao4c09045_si_004.cif
