# Supplementary material for: Tuning the Performance of Metalloporphyrin-Based Pressure-Sensitive Paints through the Nature of the Central Metal Ion
Source: ACS Omega. 2024 Dec 18;9(52):51580–90. doi: 10.1021/acsomega.4c09045 (PMC11696388; doi:10.1021/acsomega.4c09045)
Supplement: Supplementary file 1 — ao4c09045_si_001.pdf [file ao4c09045_si_001.pdf]

Tuning the performance of metalloporphyrin-based pressure-sensitive paints through the nature of the central metal ion

Elliott J. Nunn[a], Dimitrios Tsioumanis[b], George F. S. Whitehead[a], Tom B. Fisher[c], David A. Roberts[d], Mark K. Quinn[b*], Louise S. Natrajan[a*]

[a]Department of Chemistry, University of Manchester, Oxford Road, M13 9PL, United Kingdom

[b]Department of Mechanical and Aerospace Engineering, University of Manchester, Oxford Road, M13 9PL, United Kingdom

[c] BAE Systems, Warton Aerodrome, Warton PR4 1AX, United Kingdom

[d]Aircraft Research Association, Manton Lane, Bedford MK41 7PF, United Kingdom

*Email: mark.quinn@manchester.ac.uk
*Email: louise.natrajan@manchester.ac.uk

Supporting Information

Summary: 43 pages, 27 Figures including 27 Graphical Figures, 4 Schemes, 4 Tables

This document provides supporting information for this paper, including details of syntheses and characterization of compounds, crystallographic data, density functional theory calculations, luminescence data, and polystyrene PSP performance data.

Table of Contents

1. Synthesis S3
   1.1 General synthetic details S3
   1.2 Synthesis of freebase porphyrin, Fb1 ..... S3
   1.3 Synthesis of Zn1 ..... S4
   1.4 Synthesis of Ir1 ..... S4
   1.5 Synthesis of Pd1/Pt1 ..... S5
2. Characterisation Data S5
   2.1 Fb1 – (2,3,4,5,6-pentafluoro)tetraphenyl porphyrin S5
   2.2 Zn1 – Zn(II)-(2,3,4,5,6-pentafluoro)tetraphenyl porphyrin S8
   2.3 Ir1 – Ir(III)-(2,3,4,5,6-pentafluoro)tetraphenyl porphyrin S11
   2.4 Pd1 – Pd(II)-(2,3,4,5,6-pentafluoro)tetraphenyl porphyrin S13
   2.5 Pt1 – Pt(II)-(2,3,4,5,6-pentafluoro)tetraphenyl porphyrin S16
3. Computational Studies S18
   3.1 Optimised structures from DFT calculations S18
   3.2 Orbital energies and associated energy gaps S35
4. Emission spectroscopy S37
5. Polystyrene PSP performance Studies S38
6. Crystallographic data S41
7. References S43

1. Synthesis

1.1 General synthetic details

All reagents were used as purchased. Pyrrole was vacuum distilled and then stored in a fridge in the dark. All NMR Spectra were obtained using either a Bruker Avance III 500 MHz Prodigy instrument or a Bruker Avance III 400 MHz Prodigy instrument at the University of Manchester. Chemical shifts are recorded in parts per million (ppm) from high to low frequency and referenced to the residual solvent resonance. Coupling constants (J) are reported in Hertz (Hz) and splitting patterns are designated as follows: b = broad, s = singlet, d = doublet, t = triplet, q = quartet, p = pentet, m = multiplet dm = doublet of multiplets (the coupling is too small in the multiplet to discern the exact multiplicity). Mass Spectra were obtained through the Mass Spectrometry services in the department of chemistry at the University of Manchester. Column chromatography was performed using Silica gel (Sigma-Aldrich high-purity grade, pore size 60 Å, 230-400 mesh particle size, 40-63 µm particle size, for flash chromatography)

1.2 Synthesis of freebase porphyrin, Fb1

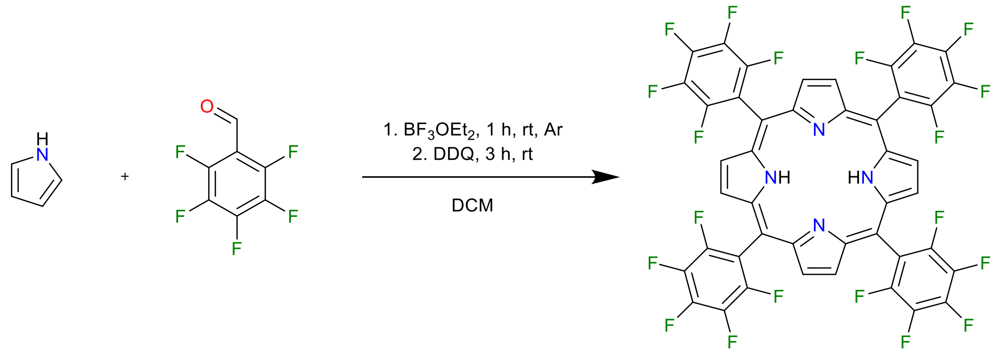

Scheme S1. The synthesis of the freebase porphyrin, **Fb1**.

To a 3 neck round bottom flask was added 2,3,4,5,6-pentafluorobenzaldehyde (1.06 g, 5.41 mmol) and dichloromethane (300 mL). This was then sealed and bubbled through with argon for 15 minutes. Freshly distilled pyrrole (0.50 mL, 5.41 mmol) was then added and the reaction stirred for 5 minutes. Then $BF_3.OEt_2$ (0.1 mL) was added and the resulting red solution stirred for 1 hour under a gentle flow of argon. After 2 hours the reaction was exposed to air and DDQ (600 mg, 2.64 mmol) was added rapidly forming a black solution. Then the reaction was stirred for 3 hours, after which the solvent was removed by rotary evaporation. The crude black residue was then purified by flash column

chromatography ($CHCl_3$ 100%) to afford a purple powder. This purple powder was recrystallized from DCM/methanol to afford a shiny purple powder **Fb1** (247 mg, 0.26 mmol, 19%)

1.3 Synthesis of Zn1

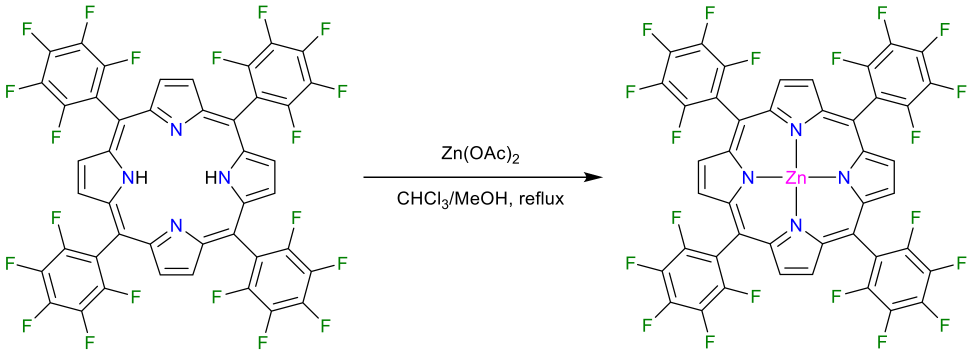

Scheme S2. The synthesis of **Zn1** from the freebase poprhyrin, **Fb1**.

Fb1 (136 mg, 0.14 mmol) was dissolved in chloroform (100 mL) and zinc acetate dehydrate (69.4 mg, 0.38 mmol) dissolved in methanol (50 mL) was then added to the purple solution. The reaction was heated at reflux overnight. Upon completion, as identified by UV-Vis absorption spectroscopy, the solution was washed with water (3 x 50 mL), dried over anhydrous magnesium sulphate and then the solvent removed via rotary evaporation to afford a pink solid **Zn1** (123 mg, 0.12 mmol, 86%).

1.4 Synthesis of Ir1

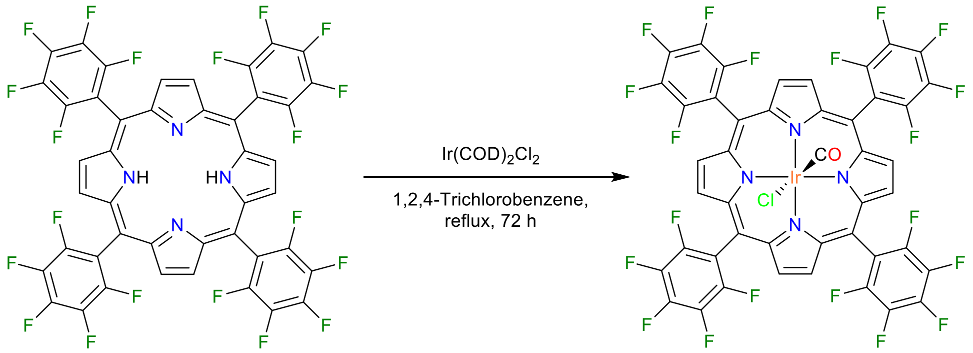

Scheme S3. The synthesis of **Ir1** from the freebase poprhyrin, **Fb1**.

Fb1 (150 mg, 0.16 mmol) and $Ir(COD)_2Cl_2$ (161.2 mg, 0.24 mmol) were dissolved in 1,2,4-trichlorobenzne (70 mL) and refluxed for 72 hours. The solvent was then removed by rotary

evaporation and the dark red residue purified by column chromatography ($CHCl_3$:hexane 50:50) to remove the fast moving purple band and then ($CHCl_3$ 100%) to remove the slower red band. This afforded a shiny red solid **Ir1** (89 mg, 0.07 mmol, 46%).

1.5 Synthesis of Pd1/Pt1

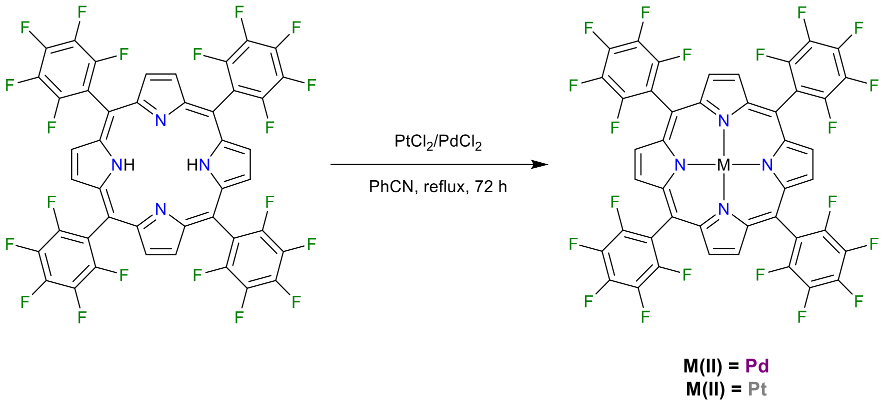

Scheme S4. The synthesis of **Pd1** and **Pt1** from the freebase poprhyrin, **Fb1**.

$PtCl_2$ /$PdCl_2$ (0.54 mmol) and PhCN (18 mL) were added to a two neck round bottom flask and then heated to 120 °C while stirring under a constant supply of argon to form a yellow solution. **Fb1** (124.74 mg, 0.13 mmol) was then added, and the reaction was refluxed for 72 hours. The solvent was then removed by vacuum distillation and the crude mixture purified by column chromatography ($CHCl_3$ 100%) to afford a violet solid **Pd1** (127 mg, 0.12 mmol, 92%) or a red solid **Pt1** (130 mg, 0.11 mmol, 87%)

2. Characterisation Data

2.1 Fb1 – (2,3,4,5,6-pentafluoro)tetraphenyl porphyrin

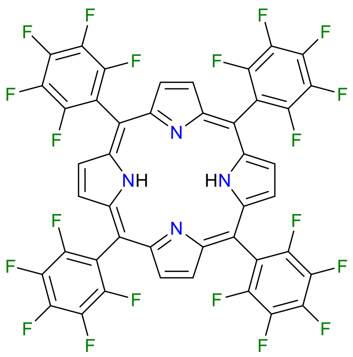

Fb1 (247 mg, 0.26 mmol, 19%). **^{1}H NMR** (500 MHz, $CDCl_3$) δ = 8.93 (s, 8H, β-pyrrole-H), δ = -2.91 (s, 2H, pyrrole-NH). **^{13}C{^{1}H} NMR** (500 MHz, $CDCl_3$) δ = 146.71 (dm, $^1J_{C\text{-}F}$ = 249.7 Hz, *o*-phenyl-C), δ = 142.44

(dm, $^{1}J_{C-F}$ = 254.4 Hz, *p*-phenyl-C), δ = 137.73 (dt, $^{1}J_{C-F}$ = 254.4 Hz, $^{2}J_{C-F}$ = 16.4 Hz *o*-phenyl-C), δ = 115.67 (td, $^{2}J_{C-F}$ = 18.5 Hz, $^{3}J_{C-F}$ = 4.0 Hz, *m*-phenyl-C), δ = 103.8 (s, β-pyrrole-C **^{19}F NMR** (400 MHz, $CDCl_3$) δ = -136.49 (dd, $^{3}J_{F-F}$ = 24.4 Hz, $^{4}J_{F-F}$ = 7.8 Hz, 8F phenyl-F), δ = -151.15 (t, $^{3}J_{F-F}$ 21.9 Hz , 4F, phenyl-F), δ = -161.26 (td, $^{3}J_{F-F}$ = 21.7 Hz, $^{4}J_{F-F}$ = 8.0 Hz, 8F, phenyl-F). **HRMS-APCI(+):** 975.0635 $[M+H]^+$, calculated for $C_{44}H_{11}N_4F_{20}$: 975.0659. **UV-Vis** [λ_{max} nm (ε $M^{-1}cm^{-1}$) in $CHCl_3$] 412 (314,900), 507 (22,300), 537 (2,800), 583 (7,100), 637 (1,200).

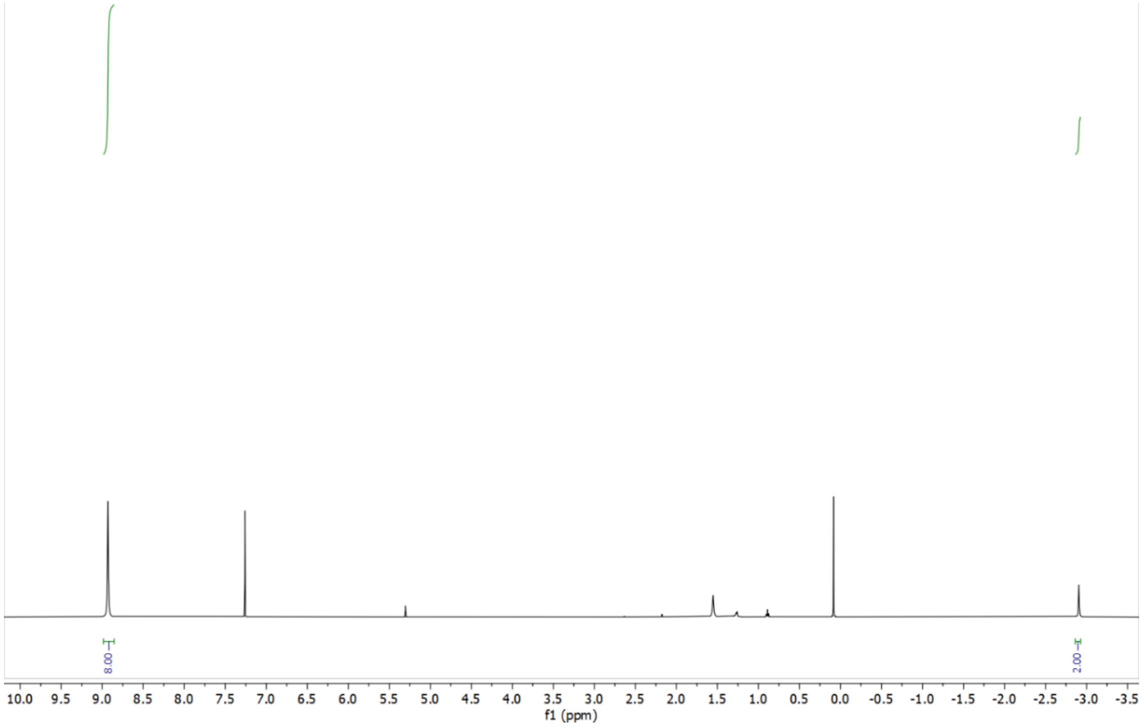

Figure S1. ^{1}H NMR spectrum of **Fb1** in $CDCl_3$.

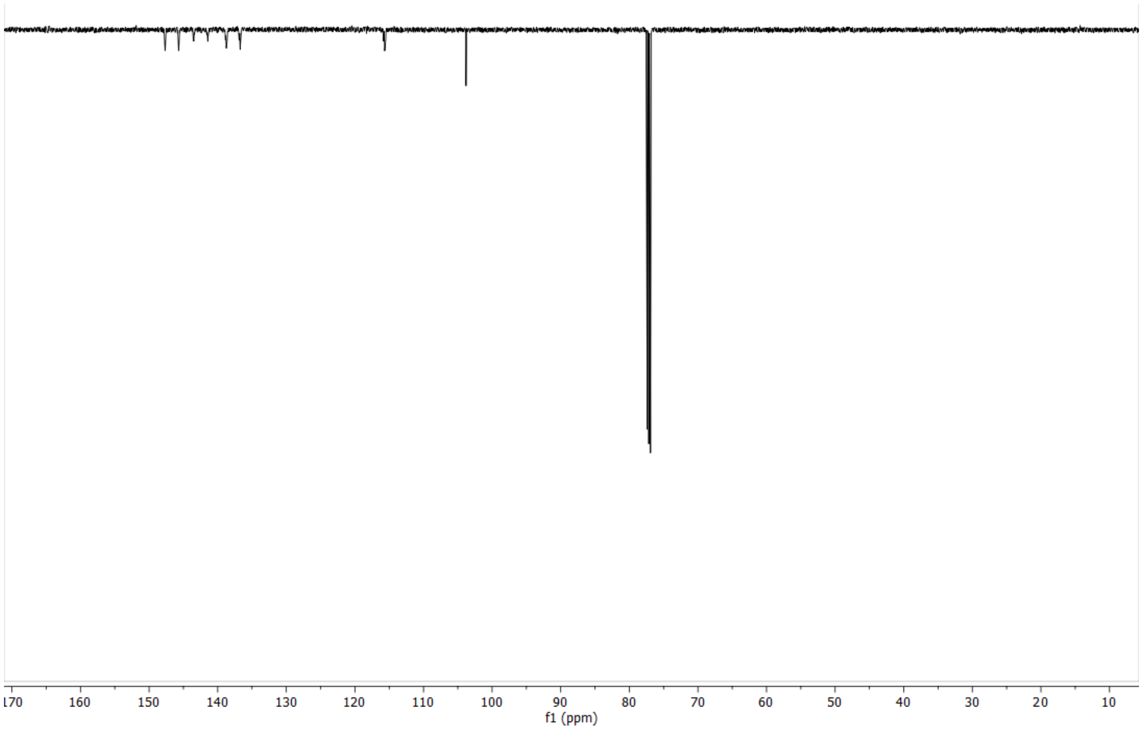

Figure S2. $^{13}C\{^{1}H\}$ DEPTQ NMR spectrum of **Fb1** in $CDCl_3$.

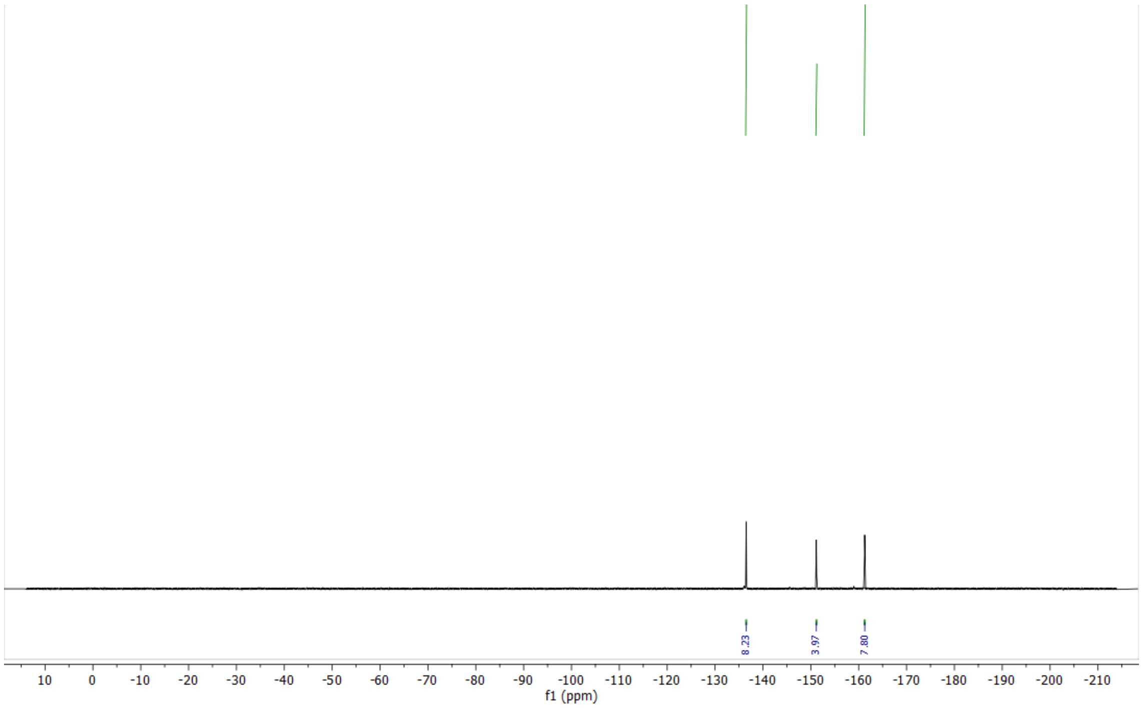

Figure S3. ^{19}F NMR spectrum of **Fb1** in $CDCl_3$.

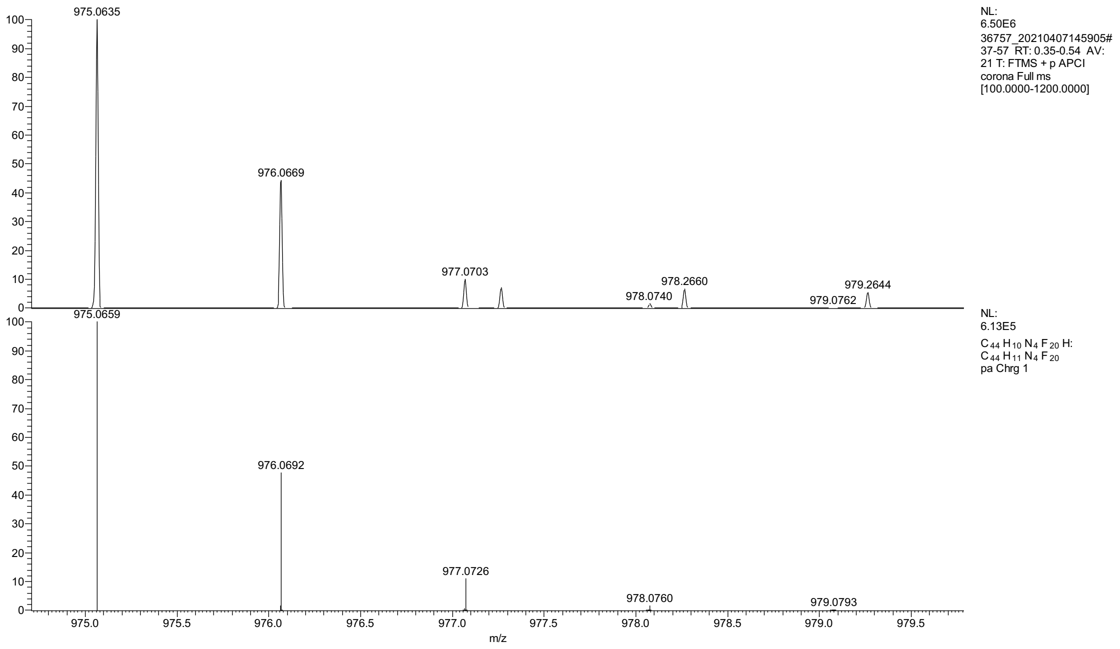

Figure S4. HRMS-ESI spectrum of **Fb1**.

2.2 Zn1 – Zn(II)-(2,3,4,5,6-pentafluoro)tetraphenyl porphyrin

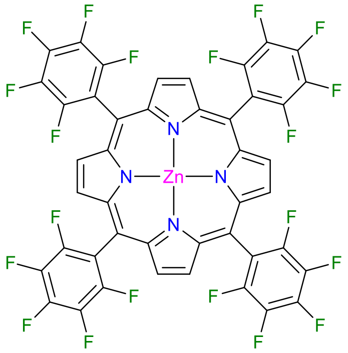

Zn1 (125 mg, 0.12 mmol, 86%). **^{1}H NMR** (500 MHz, $CDCl_3$) δ = 9.0 (s, 8H, β-pyrrole-H). **^{13}C{^{1}H} NMR** (500 MHz, $CDCl_3$) δ = 150.38 (s, α-pyrrole-C), δ = 146.67 (dm, $^{1}J_{C\text{-}F}$ = 249.6 Hz, *o*-phenyl-C), δ = 142.34 (dm, $^{1}J_{C\text{-}F}$ = 248.32 Hz, *p*-phenyl-C), δ = 137.77 (dm, $^{1}J_{C\text{-}F}$ = 249.76 Hz, *m*-phenyl-C), δ = 132.23 (s, β-pyrrole-C), δ = 116.43 (t, $^{2}J_{C\text{-}F}$ = 18.9 Hz, *i*-phenyl-C), δ = 104.40 (s, *meso*-C). **^{19}F NMR** (500 MHz, $CDCl_3$) δ = -136.78 (dd, $^{3}J_{F\text{-}F}$ = 23.6 Hz, $^{4}J_{F\text{-}F}$ = 7.8 Hz, 19F, phenyl-F), δ = -151.85 (t, $^{3}J_{F\text{-}F}$ = 21.5 Hz, $^{4}J_{F\text{-}F}$ = 7.8 Hz, 1F, phenyl-F). **HRMS-APCI(+):** 1036.9778 $[M+H]^+$, calculated for $C_{44}H_9N_4F_{20}Zn$: 1036.9794. **UV-Vis** [λ_{max} nm (ε $M^{-1}cm^{-1}$) in $CHCl_3$] 414 (468,600), 524 (22,200), 578 (3,000).

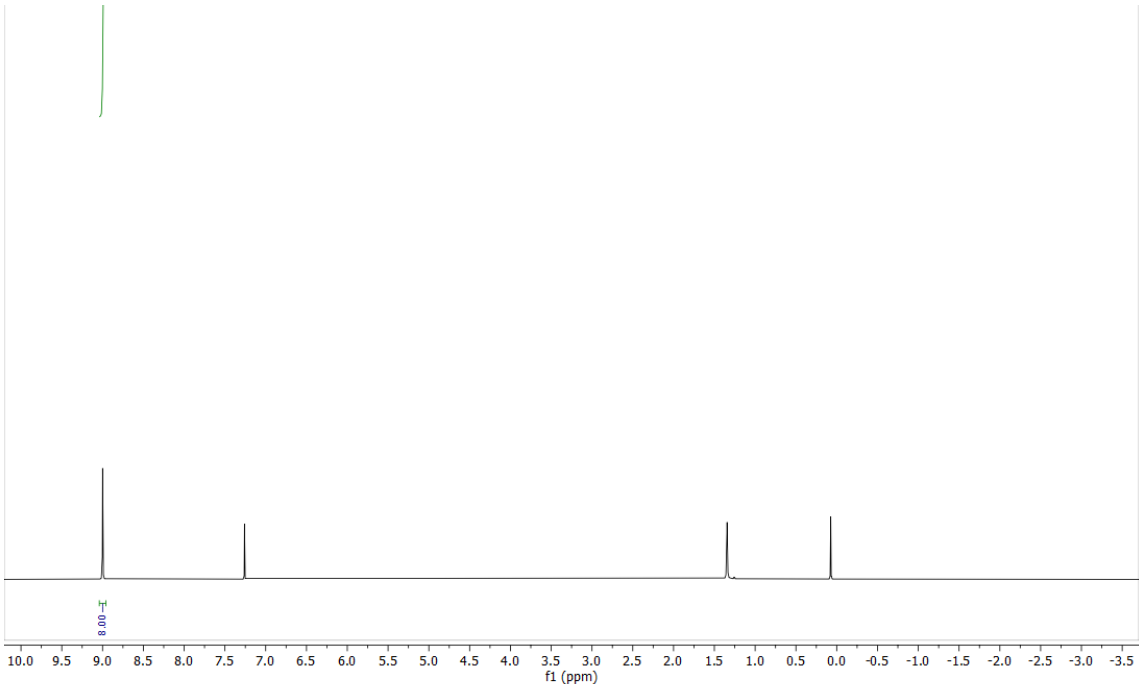

Figure S5. ^{1}H NMR spectrum of **Zn1** in $CDCl_3$.

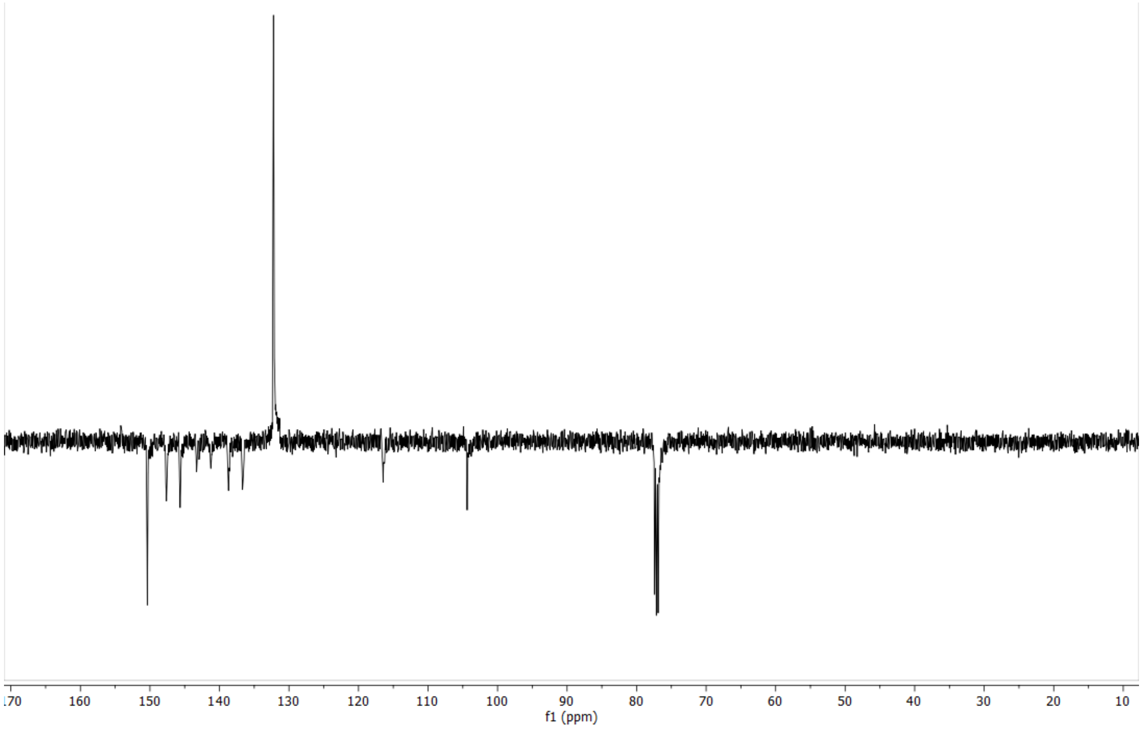

Figure S6. $^{13}C\{^{1}H\}$ DEPTQ NMR spectrum of **Zn1** in $CDCl_3$.

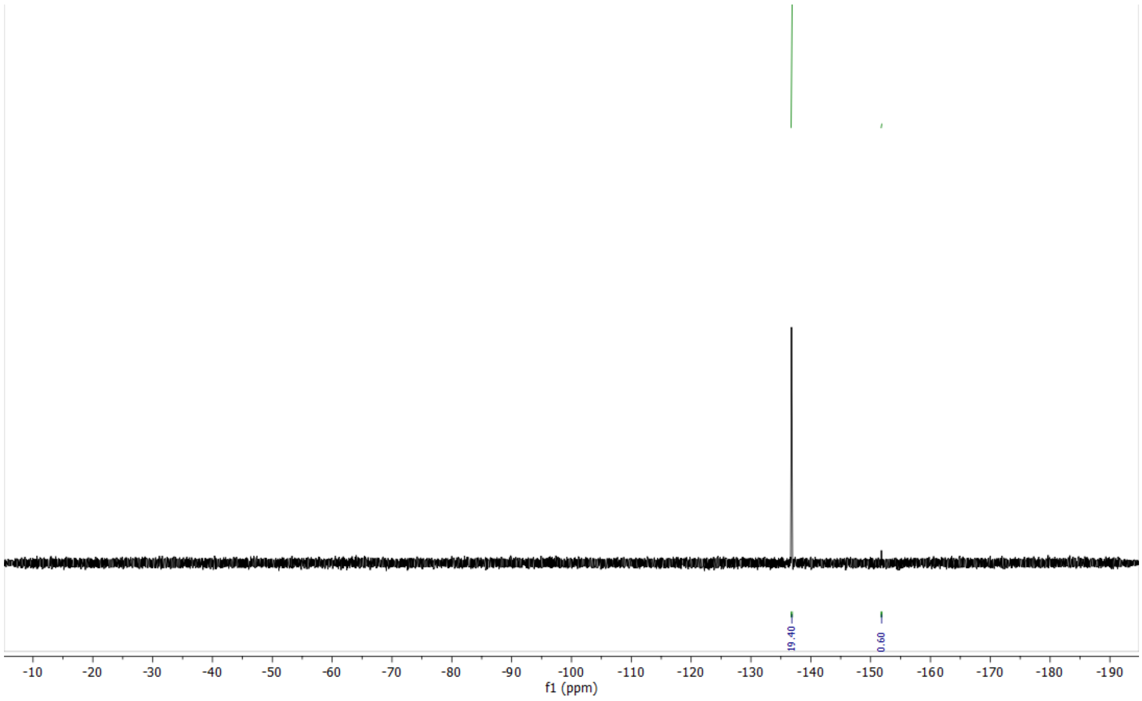

Figure S7. ^{19}F NMR spectrum of **Zn1** in $CDCl_3$

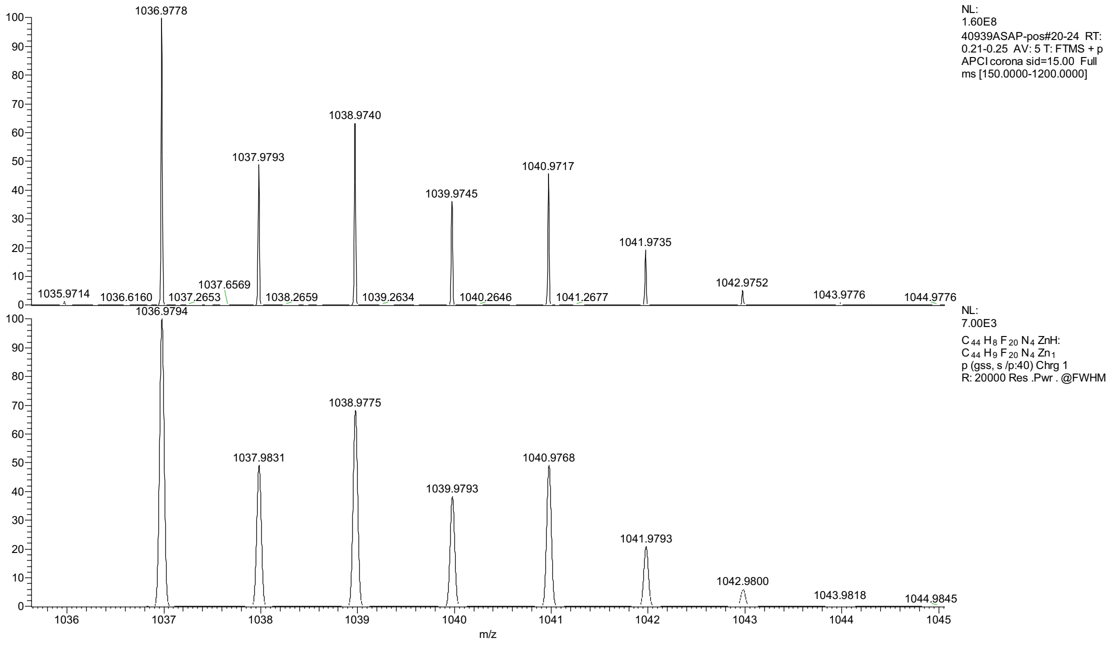

Figure S8. HRMS-APCI(+) spectrum of **Zn1**.

2.3 Ir1 – Ir(III)-(2,3,4,5,6-pentafluoro)tetraphenyl porphyrin

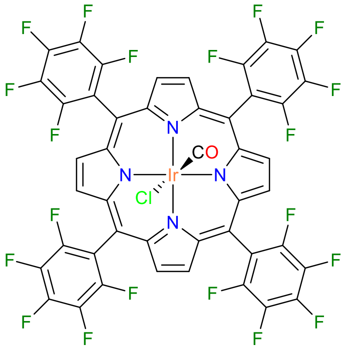

Ir1 (89 mg, 0.07 mmol, 46%). **^{1}H NMR** (400 MHz, $CDCl_3$) δ = 9.0 (s, 8H, β-pyrrole-H). **^{19}F NMR** (400 MHz, $CDCl_3$) δ = -134.70 (dm, $^3J_{C-F}$ = 23.2 Hz, 4F, phenyl-F), δ = -136.87 (dm, $^3J_{C-F}$ = 23.2 Hz, 4F, phenyl-F), δ = -150.29 (t, $^3J_{F-F}$ = 21.2 Hz, 4F, phenyl-F), δ = -160.4 (ddd, $^3J_{C-F}$ = 26.0 Hz, $^3J_{F-F}$ = 21.2 Hz, $^4J_{F-F}$ = 8.4 Hz, 4F, phenyl-F), δ = -161.09 (ddd, $^3J_{F-F}$ = 25.8 Hz, $^3J_{F-F}$ = 21.1 Hz, $^4J_{F-F}$ = 8.2 Hz, 4F, phenyl-F). **HRMS-APCI(+):** 1227.9738 $[M]^+$, calculated for $C_{45}H_8ClN_4F_{20}IrO$: 1227.9685. **FT-IR** (ATR cm^{-1}) *v* = 2055 (s, CO), *v* = 1491.34 (s, CF). **UV-Vis** [λ_{max} nm (ε $M^{-1}cm^{-1}$) in $CHCl_3$] 414 (176,400), 527 (19,100), 558 (6,000).

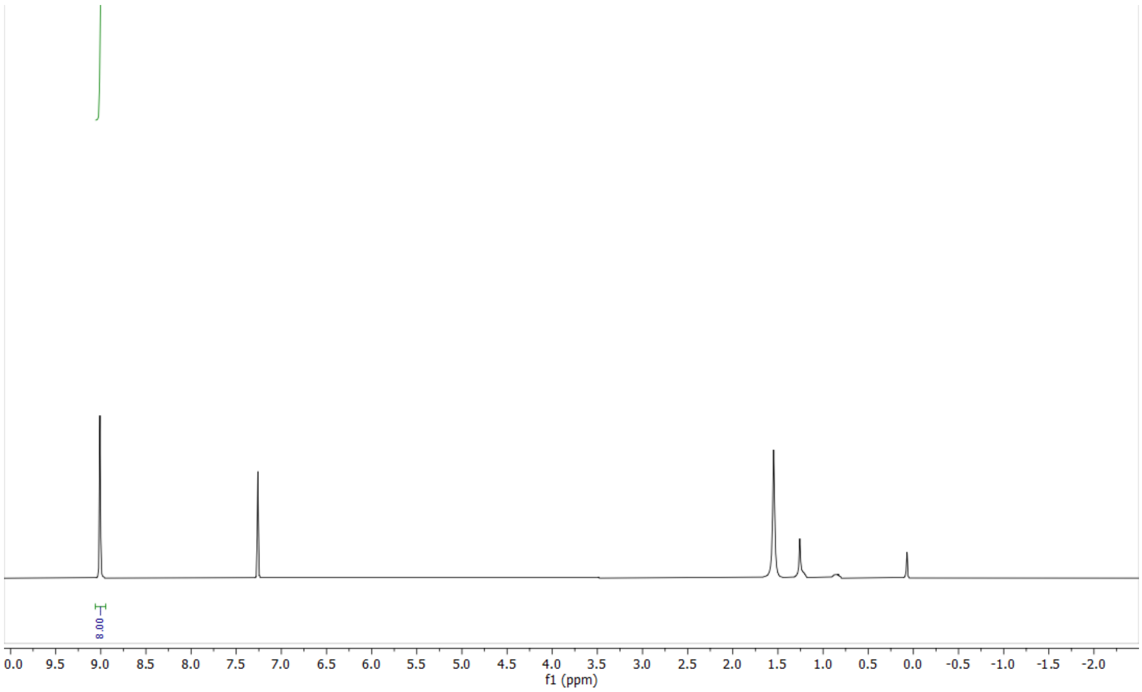

Figure S9. ^{1}H NMR spectrum of **Ir1** in $CDCl_3$.

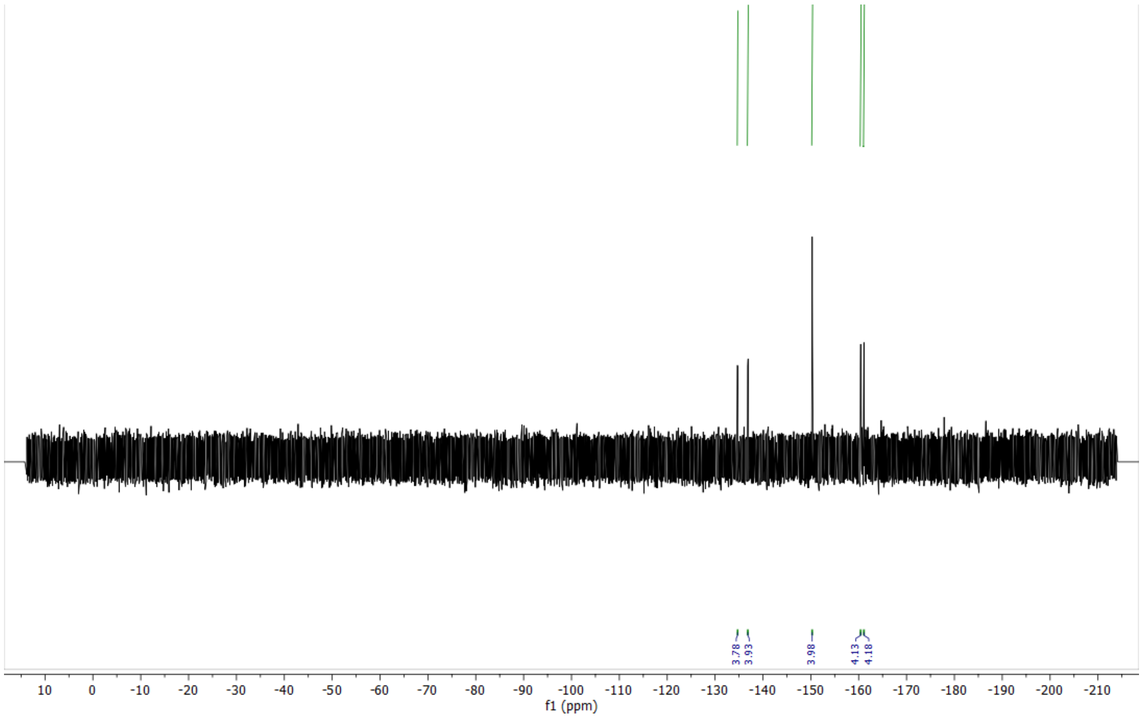

Figure S10. ^{19}F NMR spectrum of **Ir1** in $CDCl_3$.

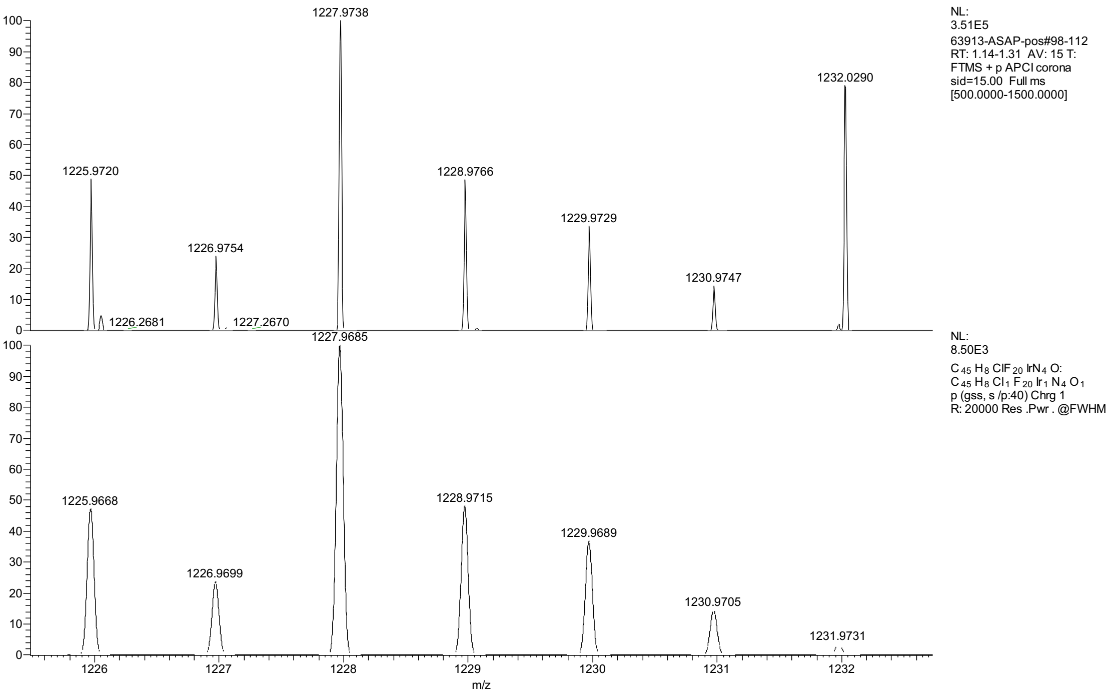

Figure S11. HRMS-APCI(+) spectrum of **Ir1**.

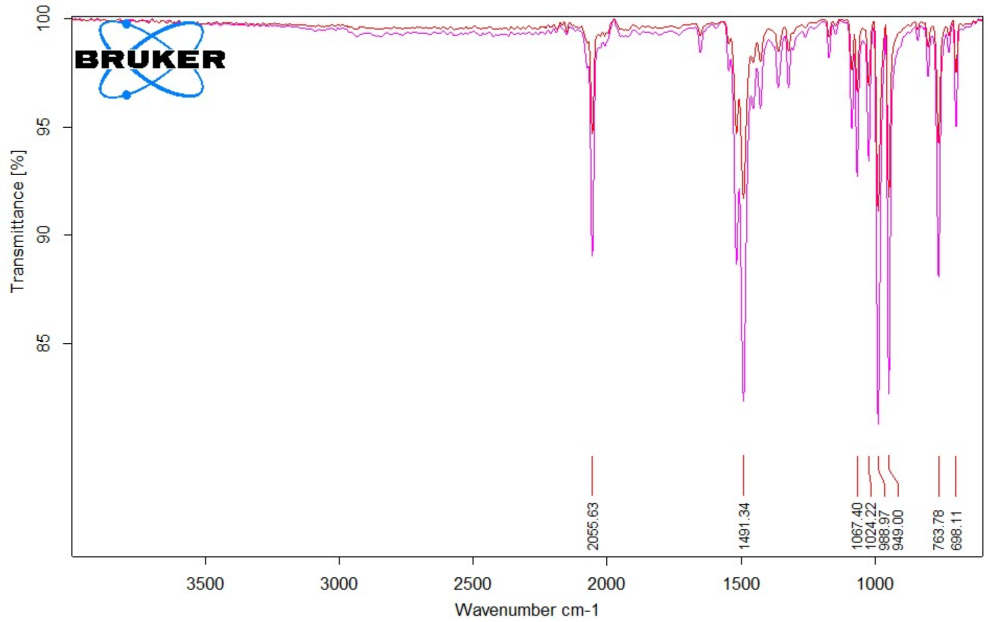

Figure S12. IR spectrum of **Ir1**.

2.4 Pd1 – Pd(II)-(2,3,4,5,6-pentafluoro)tetraphenyl porphyrin

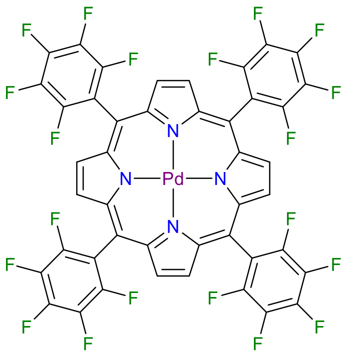

Pd1 (127 mg, 0.12 mmol, 92%). **^{1}H NMR** (400 MHz, $CDCl_3$) δ = 8.89 (s, 8H, β-pyrrole-H). **^{13}C{^{1}H} NMR** (500 MHz, $CDCl_3$) δ = 146.6 (dm, $^{1}J_{C-F}$ = 248.2 Hz, *o*-phenyl-C), δ = 142.49 (dm, $^{1}J_{C-F}$ = 256.6 Hz, *p*-phenyl-C), δ = 142.01 (s, α-pyrrole-C), δ = 137.76 (dm, $^{1}J_{C-F}$ = 253.7 Hz, *m*-phenyl-C), δ = 131.47 (s, β-pyrrole-C), δ = 115.40 (t, $^{2}J_{C-F}$ = 20.4 Hz, *i*-phenyl-C), δ = 105.74 (s, *meso*-C). **^{19}F NMR** (500 MHz, $CDCl_3$) δ = -136.42 (dd, $^{3}J_{F-F}$ = 23.1 Hz, $^{4}J_{F-F}$ = 8.0 Hz, 19F, phenyl-F), δ = -151.15 (t, $^{3}J_{F-F}$ = 20.6 Hz, 1F, phenyl-F). **HRMS-APCI(+):** 1078.9540 $[M+H]^+$, calculated for $C_{44}H_9N_4F_{20}Pd$: 1078.9554. **UV-Vis** [λ_{max} nm (ε $M^{-1}cm^{-1}$) in $CHCl_3$] 407 (271,400), 519 (22,200), 552 (18,800).

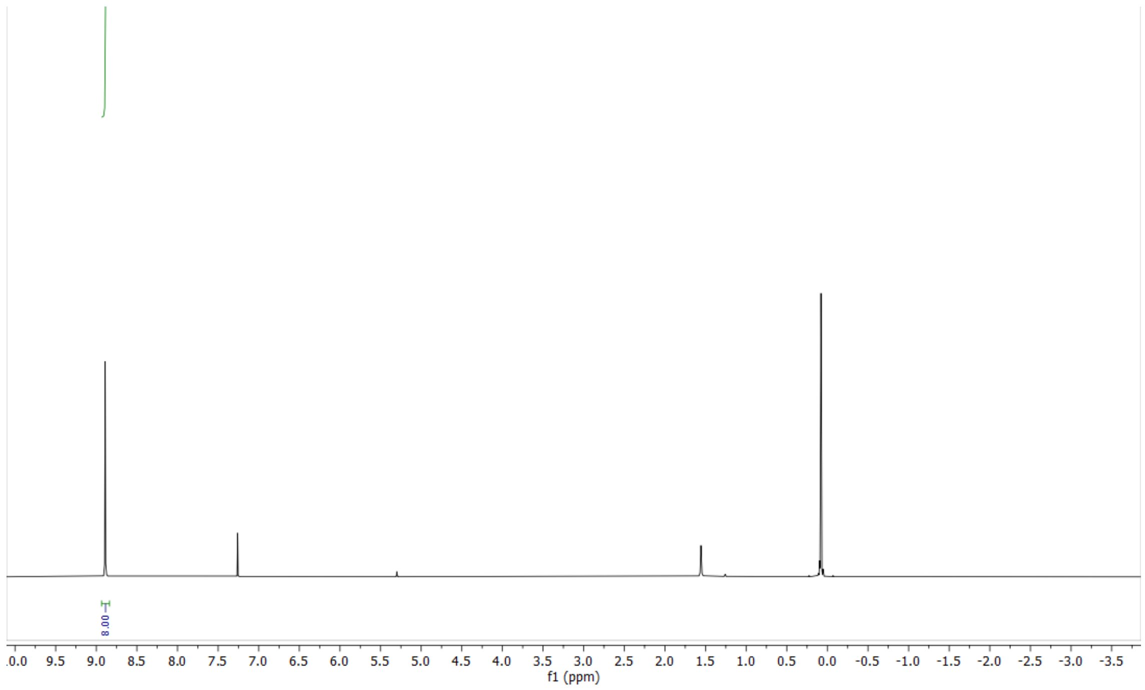

Figure S13. ^{1}H NMR spectrum of **Pd1** in $CDCl_3$.

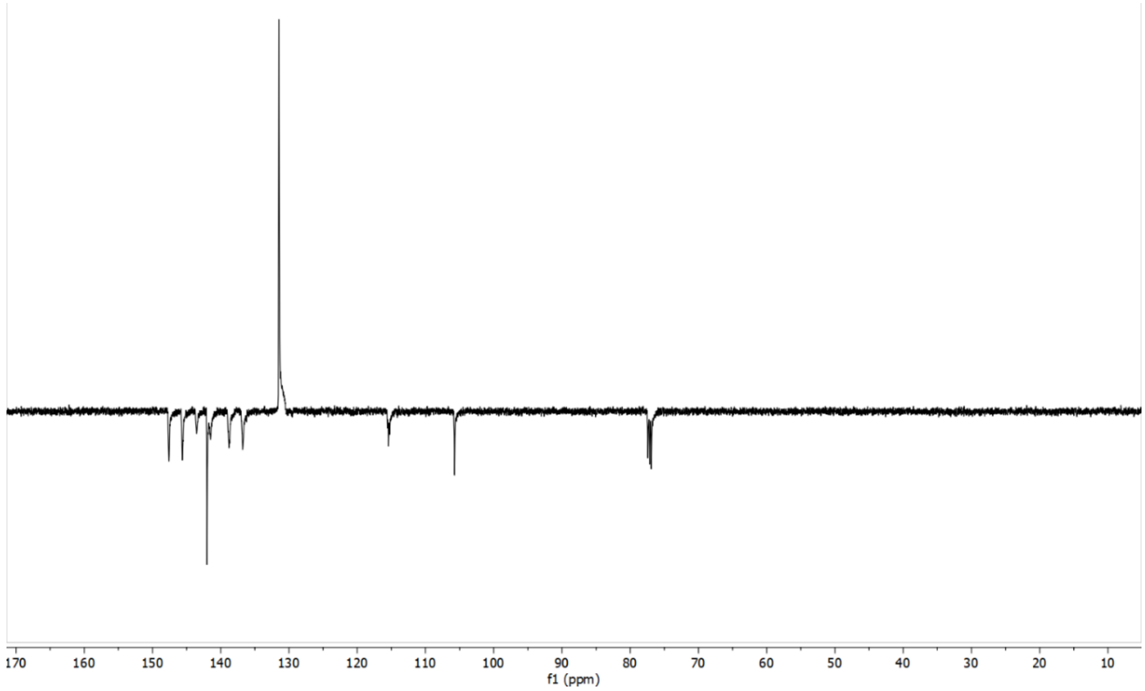

Figure S14. $^{13}C\{^{1}H\}$ DEPTQ NMR spectrum of **Pd1** in $CDCl_3$.

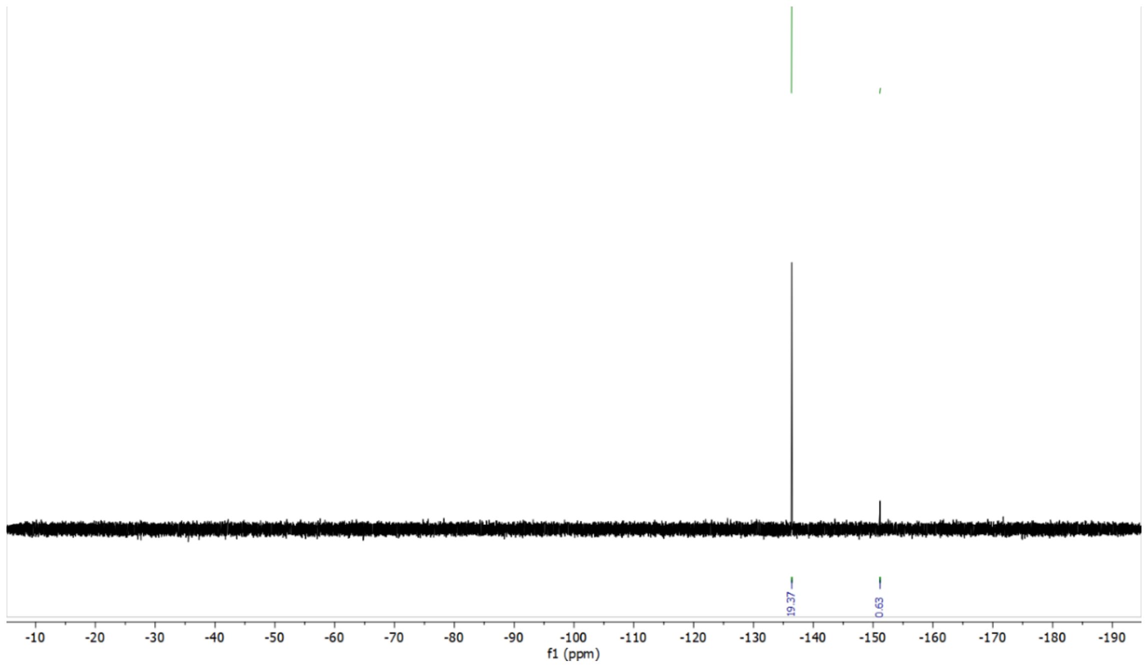

Figure S15. ^{19}F NMR spectrum of **Pd1** in $CDCl_3$.

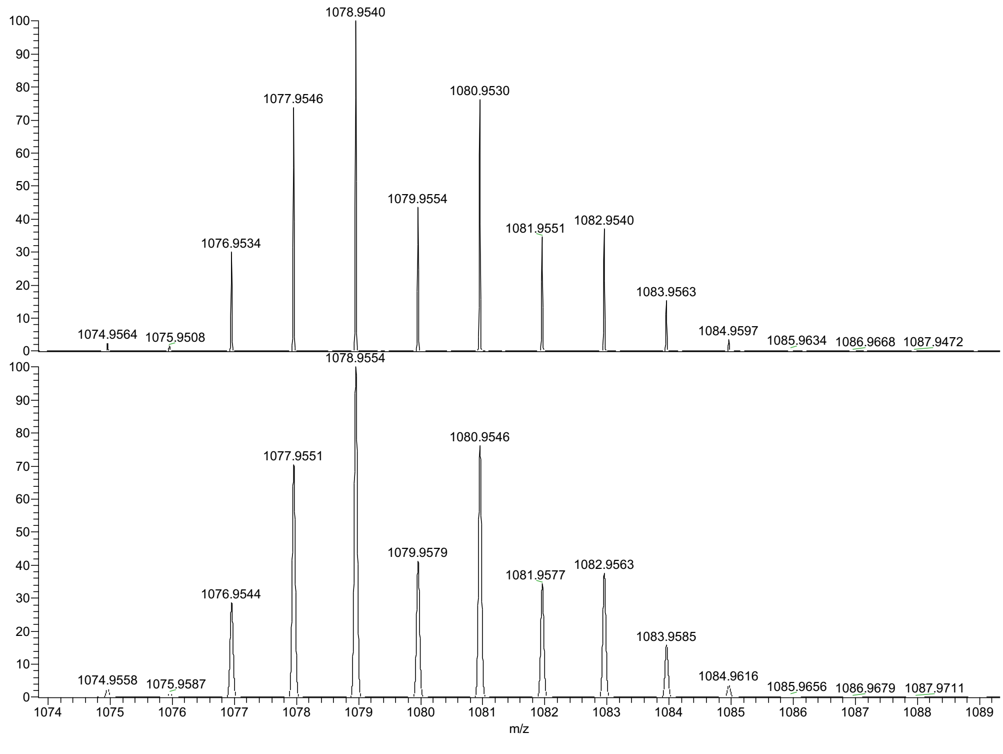

Figure S16. HRMS-APCI(+) spectrum of **Pd1**.

2.5 Pt1 – Pt(II)-(2,3,4,5,6-pentafluoro)tetraphenyl porphyrin

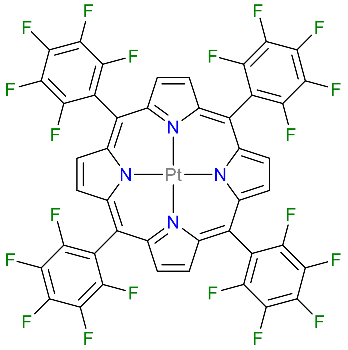

Pt1 (131.9 mg, 0.11 mmol, 87%). **^{1}H NMR** (500 MHz, $CDCl_3$) δ = 8.83 (s, $^{4}J_{H-Pt}$ = 5.0 Hz, 8H, β-pyrrole-H). **^{13}C{^{1}H} NMR** (500 MHz, $CDCl_3$) δ = 146.52 (dt, $^{1}J_{C-F}$ = 249.8 Hz, $^{3}J_{C-F}$ = 9.0 Hz *o*-phenyl-C), δ = 142.54 (dm, $^{1}J_{C-F}$ = 249.4 Hz, $^{2}J_{C-F}$ = 12.2 Hz *p*-phenyl-C), δ = 141.42 (s, α-pyrrole-C), δ = 137.77 (dd, $^{1}J_{C-F}$ = 251.1 Hz, $^{2}J_{C-F}$ = 13.2 Hz, *m*-phenyl-C), δ = 131.20 (s, $^{3}J_{C-Pt}$ = 15.5 Hz, β-pyrrole-C), δ = 114.91 (td, $^{2}J_{C-F}$ = 19.4 Hz, $^{3}J_{C-F}$ = 3.2 Hz *i*-phenyl-C), δ = 106.49 (s, *meso*-C). **^{19}F NMR** (500 MHz, $CDCl_3$) δ = -136.29 - -136.45 (m, 17F phenyl-F), δ = -151.00 (tm, $^{3}J_{F-F}$ = 20.4 Hz , 2F, phenyl-F), δ = -161.03 (td, $^{3}J_{F-F}$ = 21.9 Hz, $^{4}J_{F-F}$ = 6.8 Hz, 1F, phenyl-F). **HRMS-APCI(+):** 1168.0167 $[M+H]^+$, calculated for $C_{44}H_9N_4F_{20}Pt$: 1168.0150. **UV-Vis** [λ_{max} nm (ε $M^{-1}cm^{-1}$) in $CHCl_3$] 392 (296,500), 507 (18,800), 540 (28,400).[S1]

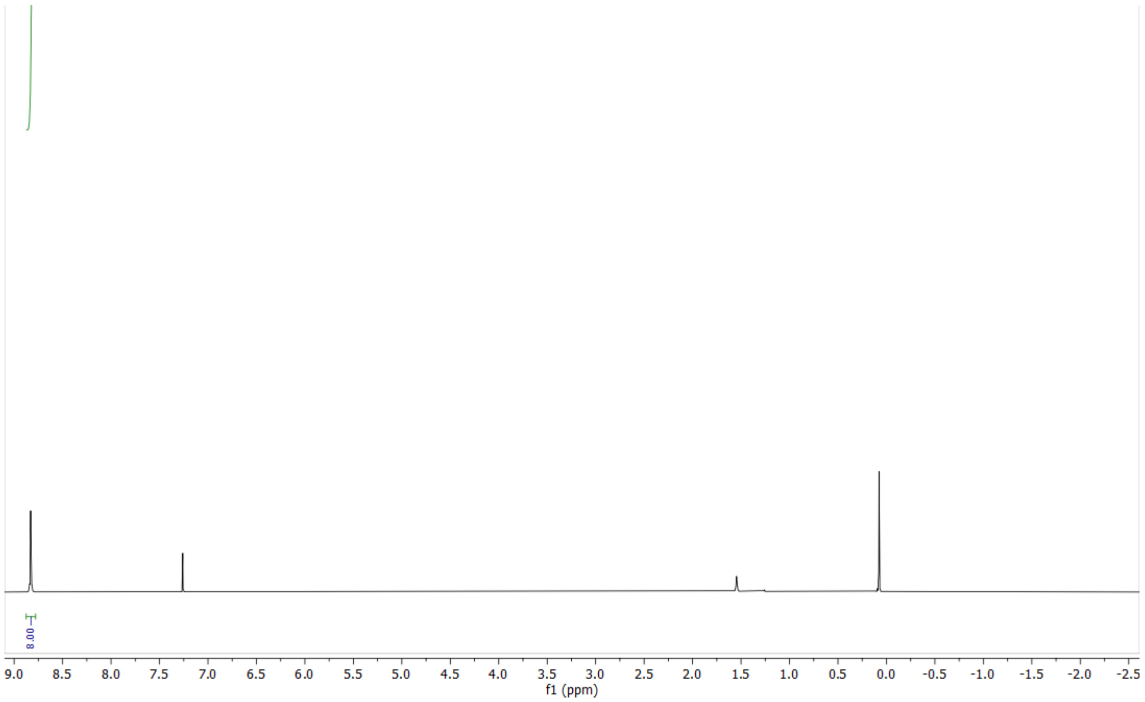

Figure S17. ^{1}H NMR spectrum of **Pt1** in $CDCl_3$.[1]

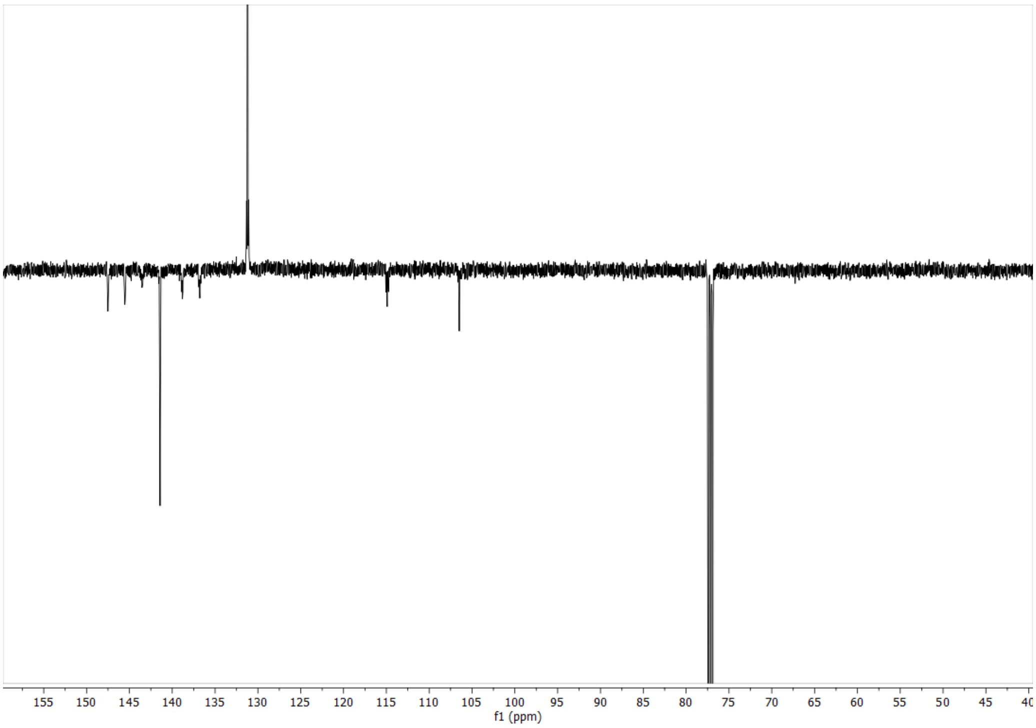

Figure S18. $^{13}C\{^{1}H\}$ DEPTQ NMR spectrum of **Pt1** in $CDCl_3$.[1]

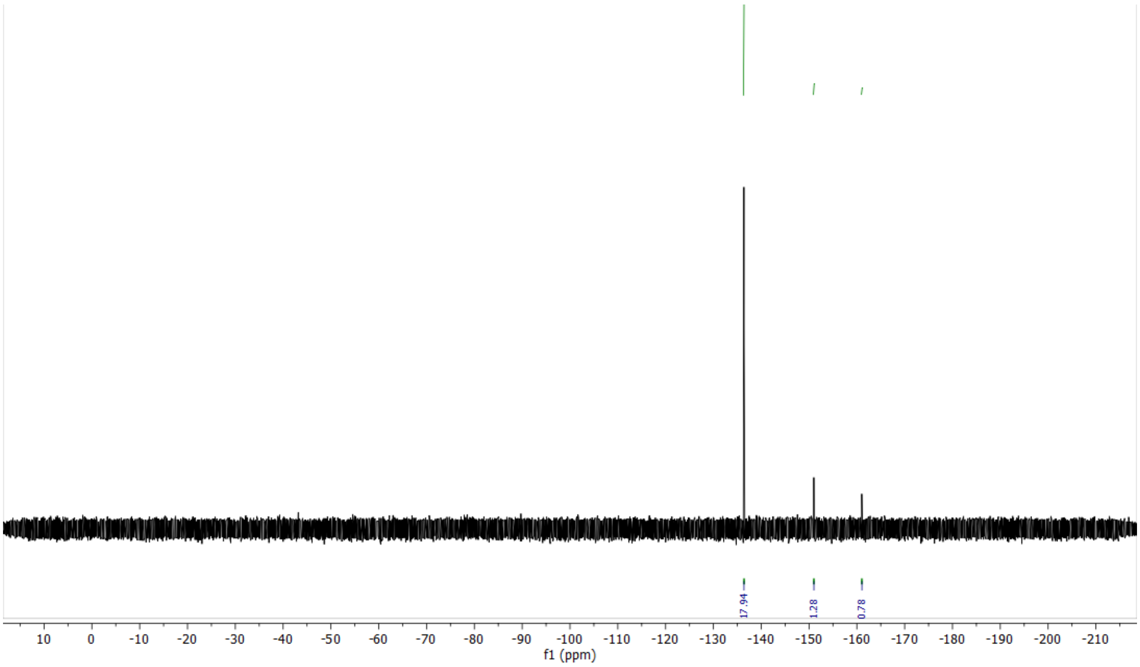

Figure S19. ^{19}F NMR spectrum of **Pt1** in $CDCl_3$.[1]

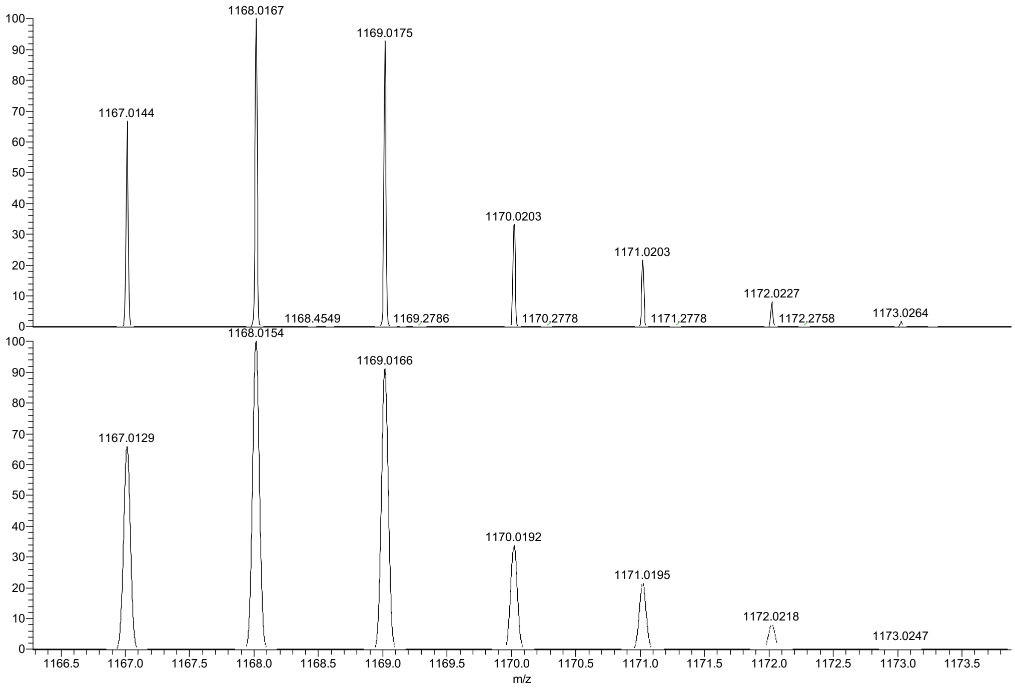

Figure S20. HRMS-APCI(+) spectrum of **Pt1**.[1]

3. Computational Studies

3.1 Optimised structures from DFT calculations

Fb1

78

C	0.025099000	0.008273000	-4.160439000
C	0.022197000	3.472132000	-0.697725000
C	0.022621000	-0.008222000	2.782850000
C	0.024641000	-3.472084000	-0.679833000
N	0.049122000	1.494069000	0.804590000
N	0.033125000	1.435584000	-2.124358000
N	0.050777000	-1.493980000	-2.182129000
N	0.033354000	-1.435538000	0.746788000

C	0.018313000	-2.858451000	-1.941893000
C	0.018471000	-1.253709000	-3.546552000
C	0.046251000	1.252010000	-3.498176000
C	0.044736000	2.809473000	-1.941288000
C	0.046010000	-2.809420000	0.563746000
C	0.045134000	-1.251944000	2.120613000
C	0.015830000	1.253760000	2.168984000
C	0.015721000	2.858513000	0.564342000
C	-0.037024000	-3.515817000	-3.227987000
C	-0.037024000	-2.539832000	-4.203929000
C	0.082311000	2.543233000	-4.198868000
C	0.081267000	3.509709000	-3.232782000
C	-0.040973000	3.515831000	1.850402000
C	-0.041039000	2.539834000	2.826336000
C	0.081940000	-3.509621000	1.855274000
C	0.081399000	-2.543135000	2.821352000
H	-0.082916000	-4.584351000	-3.377540000
H	-0.082724000	-2.689366000	-5.272470000
H	0.117699000	2.683433000	-5.269783000
H	0.115727000	4.580605000	-3.373352000
H	-0.087784000	4.584329000	1.999923000
H	-0.087694000	2.689305000	3.894846000
H	0.116955000	-4.580495000	1.995892000
H	0.115834000	-2.683281000	3.892306000
C	-0.018039000	0.021198000	-5.654586000

C	1.103274000	-0.291356000	-6.435587000
C	-1.192411000	0.347979000	-6.347833000
C	1.068964000	-0.279358000	-7.832726000
C	-1.257705000	0.368780000	-7.743178000
C	-0.119003000	0.052714000	-8.489488000
C	-0.024321000	4.966160000	-0.711164000
C	-1.201163000	5.655709000	-1.036902000
C	1.094885000	5.750642000	-0.399707000
C	-1.270840000	7.050824000	-1.057953000
C	1.056232000	7.147647000	-0.412171000
C	-0.134156000	7.800672000	-0.743222000
C	-0.022408000	-0.021196000	4.276947000
C	-1.197846000	-0.347606000	4.968561000
C	1.097927000	0.290984000	5.059481000
C	-1.265090000	-0.368399000	6.363809000
C	1.061681000	0.278939000	6.456575000
C	-0.127305000	-0.052735000	7.111692000
C	-0.020139000	-4.966159000	-0.666454000
C	1.099673000	-5.749177000	-0.979414000
C	-1.195651000	-5.657247000	-0.339199000
C	1.062836000	-7.146229000	-0.967102000
C	-1.263453000	-7.052462000	-0.318067000
C	-0.126244000	-7.800813000	-0.634447000
H	0.068661000	0.774357000	0.084426000
H	0.069311000	-0.774249000	-1.461958000

F	-2.339901000	0.657782000	-5.633271000
F	2.298905000	-0.618426000	-5.813226000
F	2.292823000	5.132266000	-0.073562000
F	-2.346860000	4.937436000	-1.344871000
F	2.294534000	0.617685000	4.438808000
F	-2.344460000	-0.656960000	4.252396000
F	-2.341790000	-4.940473000	-0.029413000
F	2.296303000	-5.129171000	-1.307250000
F	2.183718000	7.885876000	-0.103390000
F	-2.451744000	7.690936000	-1.384451000
F	-0.186605000	9.179752000	-0.758947000
F	2.198461000	-0.589502000	-8.567279000
F	-0.167265000	0.068213000	-9.868715000
F	-2.436088000	0.696477000	-8.387315000
F	-2.443005000	-7.694198000	0.010124000
F	-0.176905000	-9.179960000	-0.618748000
F	2.190808000	-7.882990000	-1.277604000
F	2.190296000	0.588652000	7.192670000
F	-0.177460000	-0.068235000	8.490854000
F	-2.444503000	-0.695687000	7.006258000

Zn1

77

C	-0.041166000	0.000001000	-5.433067000
C	-0.041158000	3.470368000	-1.962693000

C	-0.041171000	0.000000000	1.507680000
C	-0.041155000	-3.470367000	-1.962693000
N	-0.027916000	1.460390000	-0.502302000
N	-0.054414000	1.460391000	-3.423085000
N	-0.027918000	-1.460390000	-3.423085000
N	-0.054417000	-1.460390000	-0.502302000
C	-0.048980000	-2.833122000	-3.219531000
C	-0.048960000	-1.256839000	-4.795818000
C	-0.033372000	1.256840000	-4.795818000
C	-0.033341000	2.833123000	-3.219531000
C	-0.033342000	-2.833123000	-0.705855000
C	-0.033378000	-1.256840000	0.870432000
C	-0.048961000	1.256840000	0.870432000
C	-0.048980000	2.833123000	-0.705855000
C	-0.090898000	-3.516682000	-4.508108000
C	-0.090903000	-2.545421000	-5.479372000
C	0.008581000	2.545421000	-5.479372000
C	0.008584000	3.516682000	-4.508108000
C	-0.090901000	3.516682000	0.582721000
C	-0.090906000	2.545422000	1.553985000
C	0.008581000	-3.516682000	0.582721000
C	0.008575000	-2.545421000	1.553985000
H	-0.129253000	-4.586801000	-4.654008000
H	-0.129270000	-2.691328000	-6.549488000
H	0.046951000	2.691328000	-6.549489000

H	0.046948000	4.586801000	-4.654008000
H	-0.129259000	4.586801000	0.728621000
H	-0.129277000	2.691328000	2.624102000
H	0.046948000	-4.586800000	0.728621000
H	0.046945000	-2.691327000	2.624102000
C	-0.041162000	0.000000000	-6.927729000
C	1.106575000	-0.320587000	-7.666042000
C	-1.188895000	0.320583000	-7.666050000
C	1.122464000	-0.324896000	-9.063195000
C	-1.204776000	0.324886000	-9.063202000
C	-0.041154000	-0.000006000	-9.765296000
C	-0.041145000	4.965029000	-1.962693000
C	-1.188704000	5.703355000	-2.283866000
C	1.106431000	5.703329000	-1.641518000
C	-1.204562000	7.100508000	-2.288218000
C	1.122320000	7.100482000	-1.637166000
C	-0.041113000	7.802592000	-1.962692000
C	-0.041167000	0.000001000	3.002343000
C	-1.188907000	-0.320552000	3.740665000
C	1.106581000	0.320554000	3.740654000
C	-1.204787000	-0.324854000	5.137818000
C	1.122473000	0.324858000	5.137807000
C	-0.041154000	0.000003000	5.839909000
C	-0.041137000	-4.965028000	-1.962694000
C	1.106438000	-5.703326000	-2.283876000

C	-1.188694000	-5.703356000	-1.641515000
C	1.122329000	-7.100479000	-2.288230000
C	-1.204549000	-7.100510000	-1.637164000
C	-0.041101000	-7.802591000	-1.962698000
Zn	-0.041167000	0.000000000	-1.962693000
F	2.279560000	5.034314000	-1.322350000
F	-2.361848000	5.034367000	-2.603035000
F	-2.359405000	7.791126000	-2.608274000
F	2.277178000	7.791074000	-1.317107000
F	-0.041098000	9.183393000	-1.962691000
F	2.279920000	0.638990000	3.071656000
F	-2.362252000	-0.638991000	3.071679000
F	-2.359832000	-0.644204000	5.828425000
F	-0.041148000	0.000005000	7.220710000
F	2.277525000	0.644210000	5.828403000
F	2.279563000	-5.034309000	-2.603052000
F	2.277186000	-7.791068000	-2.608297000
F	-0.041083000	-9.183392000	-1.962700000
F	-2.359388000	-7.791130000	-1.317100000
F	-2.361836000	-5.034370000	-1.322337000
F	-2.362229000	0.639060000	-6.997061000
F	2.279904000	-0.639062000	-6.997045000
F	2.277504000	-0.644285000	-9.753792000
F	-2.359813000	0.644273000	-9.753808000
F	-0.041150000	-0.000009000	-11.146097000

Ir1

80

C	0.094949000	-1.032091000	-2.997560000
C	0.081230000	2.433091000	0.468156000
C	0.080070000	-1.033345000	3.933949000
C	0.091568000	-4.498751000	0.468478000
N	0.105977000	0.421227000	1.922352000
N	0.113851000	0.422096000	-0.986319000
N	0.117714000	-2.486716000	-0.985742000
N	0.112424000	-2.487653000	1.922833000
C	0.078840000	-3.856549000	-0.776253000
C	0.080582000	-2.276916000	-2.355572000
C	0.111398000	0.213204000	-2.357016000
C	0.104820000	1.792641000	-0.777180000
C	0.108494000	-3.858321000	1.713841000
C	0.103286000	-2.278611000	3.293397000
C	0.063931000	0.211282000	3.291945000
C	0.064567000	1.790873000	1.712687000
C	0.007477000	-4.530622000	-2.060811000
C	0.008743000	-3.561280000	-3.029983000
C	0.117437000	1.499231000	-3.032309000
C	0.113274000	2.468338000	-2.062985000
C	-0.014604000	2.464800000	2.996966000
C	-0.014847000	1.495435000	3.966141000

C	0.113604000	-4.533704000	2.999829000
C	0.110237000	-3.564460000	3.969012000
H	-0.053770000	-5.599593000	-2.201642000
H	-0.051294000	-3.701863000	-4.099056000
H	0.129580000	1.642252000	-4.102645000
H	0.121483000	3.538766000	-2.205534000
H	-0.079147000	3.533644000	3.137349000
H	-0.079475000	1.635669000	5.035004000
H	0.124548000	-5.604063000	3.142765000
H	0.117970000	-3.707183000	5.039426000
C	0.082529000	-1.034627000	-4.490392000
C	1.229112000	-1.349144000	-5.232491000
C	-1.073285000	-0.724831000	-5.220188000
C	1.236841000	-1.357724000	-6.629613000
C	-1.096833000	-0.725152000	-6.617437000
C	0.065826000	-1.043463000	-7.325062000
C	0.062548000	3.925868000	0.470623000
C	-1.096369000	4.650381000	0.160335000
C	1.205848000	4.673045000	0.785241000
C	-1.126066000	6.047523000	0.160259000
C	1.207487000	6.070175000	0.793226000
C	0.033418000	6.760314000	0.478515000
C	0.060734000	-1.031072000	5.426709000
C	-1.097144000	-1.345970000	6.150805000
C	1.202509000	-0.712013000	6.174299000

C	-1.127227000	-1.346595000	7.547945000
C	1.203574000	-0.703788000	7.571431000
C	0.030632000	-1.023502000	8.261168000
C	0.075529000	-5.991540000	0.465860000
C	1.219910000	-6.736834000	0.150869000
C	-1.082228000	-6.718108000	0.776111000
C	1.223688000	-8.133982000	0.142353000
C	-1.109711000	-8.115267000	0.775861000
C	0.050854000	-8.826140000	0.457103000
F	-2.241353000	-0.411676000	-4.542819000
F	2.405756000	-1.658568000	-4.566345000
F	2.389076000	-1.670940000	-7.325647000
F	0.057803000	-1.047255000	-8.704746000
F	-2.256659000	-0.415558000	-7.301886000
F	-2.248281000	-6.037387000	1.089536000
F	-2.271312000	-8.796424000	1.086023000
F	0.039005000	-10.205800000	0.453354000
F	2.373836000	-8.833319000	-0.171233000
F	2.398310000	-6.074076000	-0.159089000
F	2.380730000	-0.396786000	5.513759000
F	2.351048000	-0.385866000	8.273152000
F	0.016049000	-1.020384000	9.640803000
F	-2.288765000	-1.662052000	8.226755000
F	-2.260401000	-1.664239000	5.467559000
F	2.385304000	4.012141000	1.095077000

F	-2.261309000	3.967563000	-0.152684000
F	2.356636000	6.771500000	1.105979000
F	-2.288798000	6.726733000	-0.149929000
F	0.019341000	8.139959000	0.481755000
Cl	-2.292012000	-1.037291000	0.462647000
Ir	0.184617000	-1.032665000	0.468398000
C	2.048658000	-1.029258000	0.472772000
O	3.219973000	-1.027108000	0.475843000

Pd1

77

C	0.183462000	-1.067617000	-3.006690000
C	0.182145000	2.394326000	0.457351000
C	0.181180000	-1.069731000	3.919335000
C	0.186159000	-4.531708000	0.455163000
N	0.181542000	0.374203000	1.900206000
N	0.183514000	0.375325000	-0.986707000
N	0.183748000	-2.511820000	-0.987611000
N	0.184025000	-2.512422000	1.899212000
C	0.163349000	-3.885706000	-0.786223000
C	0.161892000	-2.309347000	-2.361343000
C	0.205303000	0.173725000	-2.360572000
C	0.204819000	1.749084000	-0.784435000
C	0.207141000	-3.886183000	1.696796000
C	0.204588000	-2.310997000	3.273135000

C	0.159038000	0.171975000	3.273970000
C	0.159607000	1.748074000	1.698570000
C	0.114658000	-4.563674000	-2.071738000
C	0.113640000	-3.594439000	-3.040224000
C	0.254413000	1.459221000	-3.038628000
C	0.254185000	2.427835000	-2.069517000
C	0.109437000	2.426148000	2.984012000
C	0.108985000	1.457068000	3.952645000
C	0.256419000	-4.564972000	2.981892000
C	0.254678000	-3.596475000	3.951097000
H	0.074466000	-5.633828000	-2.212682000
H	0.072480000	-3.734656000	-4.110437000
H	0.295692000	1.600094000	-4.108748000
H	0.295167000	3.498045000	-2.209789000
H	0.067909000	3.496280000	3.124740000
H	0.067045000	1.597273000	5.022828000
H	0.298737000	-5.635142000	3.122056000
H	0.295360000	-3.737275000	5.021246000
C	0.183330000	-1.067165000	-4.499943000
C	1.332666000	-1.384161000	-5.236963000
C	-0.966191000	-0.749852000	-5.236520000
C	1.348253000	-1.388199000	-6.634235000
C	-0.982136000	-0.745154000	-6.633791000
C	0.182967000	-1.066508000	-7.335723000
C	0.181945000	3.887579000	0.457865000

C	-0.967618000	4.624380000	0.141242000
C	1.331399000	4.624353000	0.774978000
C	-0.983484000	6.021654000	0.137313000
C	1.347088000	6.021622000	0.779710000
C	0.181757000	6.723346000	0.458700000
C	0.179353000	-1.070294000	5.412588000
C	-0.971367000	-1.387063000	6.147565000
C	1.327899000	-0.754039000	6.151172000
C	-0.989228000	-1.391875000	7.544801000
C	1.341620000	-0.750342000	7.548469000
C	0.175166000	-1.071380000	8.248327000
C	0.189263000	-6.024952000	0.454519000
C	1.340635000	-6.758714000	0.137345000
C	-0.958509000	-6.764776000	0.770601000
C	1.359875000	-8.155923000	0.131964000
C	-0.970823000	-8.162100000	0.773872000
C	0.196245000	-8.860718000	0.452415000
F	2.503469000	3.953677000	1.091215000
F	2.502093000	6.711912000	1.096266000
F	0.181656000	8.103428000	0.459130000
F	-2.138604000	6.711975000	-0.178758000
F	-2.139648000	3.953752000	-0.175269000
F	-2.132336000	-6.097332000	1.087153000
F	-2.124269000	-8.855528000	1.089290000
F	0.199579000	-10.240797000	0.451360000

F	2.516688000	-8.843093000	-0.184744000
F	2.511069000	-6.084888000	-0.178294000
F	2.501083000	-0.437790000	5.482433000
F	-2.142578000	-1.702627000	5.475033000
F	-2.145488000	-1.707890000	8.233250000
F	0.173137000	-1.071824000	9.628412000
F	2.495811000	-0.434834000	8.240636000
F	-2.138093000	-0.433363000	-4.565663000
F	-2.137217000	-0.428413000	-7.323870000
F	0.182769000	-1.066277000	-8.715806000
F	2.503109000	-1.704760000	-7.324771000
F	2.504709000	-1.701073000	-4.566553000
Pd	0.183303000	-1.068736000	0.456721000

Pt1

77

C	4.821677000	0.195904000	-4.503003000
C	4.817209000	3.656772000	-1.040826000
C	4.818801000	0.194544000	2.420040000
C	4.820009000	-3.266392000	-1.042195000
N	4.813038000	1.634495000	0.398316000
N	4.824260000	1.635063000	-2.480771000
N	4.815163000	-1.244093000	-2.481294000
N	4.824193000	-1.244645000	0.397780000
C	4.795578000	-2.621143000	-2.281859000

C	4.796559000	-1.043960000	-3.858252000
C	4.845406000	1.435585000	-3.857801000
C	4.843005000	3.012029000	-2.280679000
C	4.844338000	-2.621599000	0.197679000
C	4.844016000	-1.045128000	1.774821000
C	4.793440000	1.434403000	1.775260000
C	4.792472000	3.011545000	0.198826000
C	4.748501000	-3.296760000	-3.566417000
C	4.749046000	-2.328133000	-4.534567000
C	4.893301000	2.720101000	-4.533426000
C	4.891845000	3.688285000	-3.564823000
C	4.743883000	3.687153000	1.483338000
C	4.744381000	2.718542000	2.451511000
C	4.892870000	-3.297805000	1.481887000
C	4.892584000	-2.329623000	2.450481000
H	4.708730000	-4.366864000	-3.706953000
H	4.709933000	-2.468160000	-5.604760000
H	4.934115000	2.860727000	-5.603480000
H	4.931231000	4.758483000	-3.704768000
H	4.703182000	4.757216000	1.623902000
H	4.704263000	2.858461000	3.521684000
H	4.933250000	-4.367955000	1.621872000
H	4.932750000	-2.470171000	3.520565000
C	4.823895000	0.196045000	-5.996162000
C	5.975394000	-0.118755000	-6.730512000

C	3.674976000	0.510900000	-6.734503000
C	5.993466000	-0.123216000	-8.127735000
C	3.661792000	0.515453000	-8.131812000
C	4.828843000	0.196161000	-8.831430000
C	4.816152000	5.149940000	-1.040649000
C	3.665177000	5.885359000	-1.354866000
C	5.966016000	5.887188000	-0.726588000
C	3.648426000	7.282619000	-1.359312000
C	5.980569000	7.284455000	-0.722197000
C	4.813918000	7.985181000	-1.040749000
C	4.819253000	0.194362000	3.913199000
C	3.669365000	-0.120111000	4.650166000
C	5.969966000	0.508930000	4.648943000
C	3.654497000	-0.124569000	6.047464000
C	5.986400000	0.513334000	6.046181000
C	4.820829000	0.194347000	6.748482000
C	4.820770000	-4.759547000	-1.042552000
C	5.971565000	-5.495099000	-1.357261000
C	3.670937000	-5.496687000	-0.728245000
C	5.988111000	-6.892341000	-1.362127000
C	3.656209000	-6.893972000	-0.724214000
C	4.822598000	-7.594807000	-1.043383000
F	2.501821000	0.825866000	-6.065523000
F	7.146097000	-0.433762000	-6.057266000
F	7.149749000	-0.438320000	-8.816379000

F	4.831267000	0.196160000	-10.211424000
F	2.507788000	0.830271000	-8.824463000
F	7.138797000	5.216975000	-0.412658000
F	7.135469000	7.976012000	-0.408413000
F	4.812775000	9.365183000	-1.040983000
F	2.492568000	7.972422000	-1.673722000
F	2.493632000	5.213301000	-1.669219000
F	7.141612000	0.823655000	3.977162000
F	2.496830000	-0.434463000	3.979790000
F	7.142008000	0.828116000	6.736204000
F	2.499486000	-0.438795000	6.738656000
F	4.821625000	0.194518000	8.128479000
F	2.498474000	-4.826406000	-0.413509000
F	7.143229000	-4.823147000	-1.671552000
F	7.143841000	-7.582150000	-1.676843000
F	4.823413000	-8.974805000	-1.043791000
F	2.501414000	-7.585496000	-0.409894000
Pt	4.818832000	0.195237000	-1.041516000

3.2 Orbital energies and associated energy gaps

Table S1. The MO energies of the optimised structures from DFT calculations in chloroform with some highlighted energy gaps in eV and the average M-N bond distance. The abbreviations are as follow HOMO-1 (H-1), HOMO (H), LUMO (L) and LUMO+1 (L+1).

Porphyrin	H-1	H	L	L+1	H to H-1 gap	H-1 to L gap	H to L gap	Average M-N bond distance from DFT (Å)
**Fb1**	-6.24	-6.13	-3.29	-3.28	0.11	2.95	2.84	-
**Zn1**	-6.08	-6.05	-3.14	-3.14	0.03	2.94	2.91	2.065
**Ir1**	-6.41	-6.40	-3.37	-3.37	0.01	3.05	3.04	2.058
**Pd1**	-6.32	-6.24	-3.20	-3.20	0.08	3.12	3.04	2.041
**Pt1**	-6.40	-6.27	-3.18	-3.18	0.13	3.23	3.09	2.036

Table S2. The MO energies of the excited state structures from TD-DFT calculations in chloroform with some highlighted energy gaps in eV. The abbreviations are as follow HOMO-1 (H-1), SOMO-HOMO (SH), SOMO-LUMO (SL) and LUMO+1 (L+1).

Porphyrin	H-1	SH	SL	L+1	H to H-1 gap	H-1 to L gap	H to L gap
**Fb1**	-7.26	-7.25	-2.60	-2.59	0.01	4.66	4.65
**Zn1**	-7.13	-7.06	-2.41	-2.41	0.06	4.72	4.66
**Ir1**	-7.53	-7.45	-2.67	-2.67	0.08	4.86	4.78
**Pd1**	-7.44	-7.28	-2.50	-2.50	0.16	4.94	4.78
**Pt1**	-7.54	-7.32	-2.47	-2.47	0.22	5.07	4.85

Table S3. Excited state compositions and predicted absorption properties from TD-DFT calculations of **Fb1**, **Zn1**, **Pt1**, **Pd1** and **Ir1**. MOs are abbreviated as follows, HOMO-1 (H-1), HOMO (H), LUMO (L) and LUMO (L+1).

Porphyrin	Excited state	% contribution of the electronic transition to the excited state				λ (nm)	Oscillator strength f
		H-1 to L	H-1 to L+1	H to L	H to L+1		
**Fb1**	Q_x		45	53		585.2	0
	Q_y	51			47	528.9	0
	B_x		50	38		391.7	1.548
	B_y	47			51	380.7	1.987
**Zn1**	Q_x	45			52	545.6	0.001
	Q_y		45	52		545.6	0.001
	B_x	50			46	382.2	1.863
	B_y		50	46		382.2	1.863
**Ir1**	Q_x	45			52	518.6	0.003
	Q_y		45	52		518.6	0.003
	B_x		51	44		379.6	1.514
	B_y	51			44	379.5	1.514
**Pd1**	Q_x	11	28	42	17	510.6	0.010
	Q_y	28	11	17	42	510.6	0.010
	B_x^1	2	27	19	1	372.1	0.856
	B_y	53	4	3	36	372.1	1.670
	B_x^2	3	25	17	2	372.1	0.815
**Pt1**	Q_x	29	7	12	5	492.4	0.021
	Q_y	7	29	5	12	492.4	0.021
	B_x	59	2	1	35	361.8	1.724
	B_y	2	59	35	1	361.8	1.724

4. Emission spectroscopy

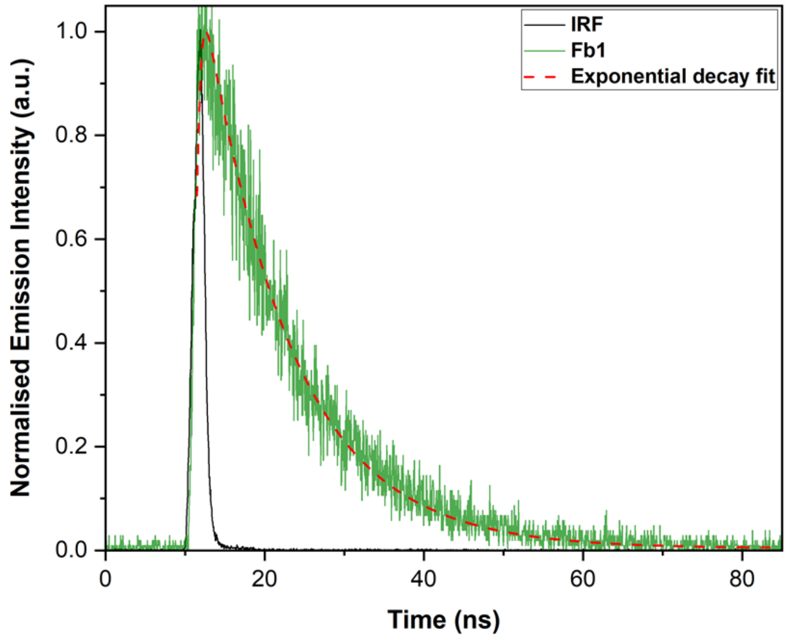

Figure S21. The lifetime of emission for **Fb1** in deoxygenated chloroform at a concentration of 0.5 μM, fitted to a mono-exponential decay with an IRF via reconvolution fitting.

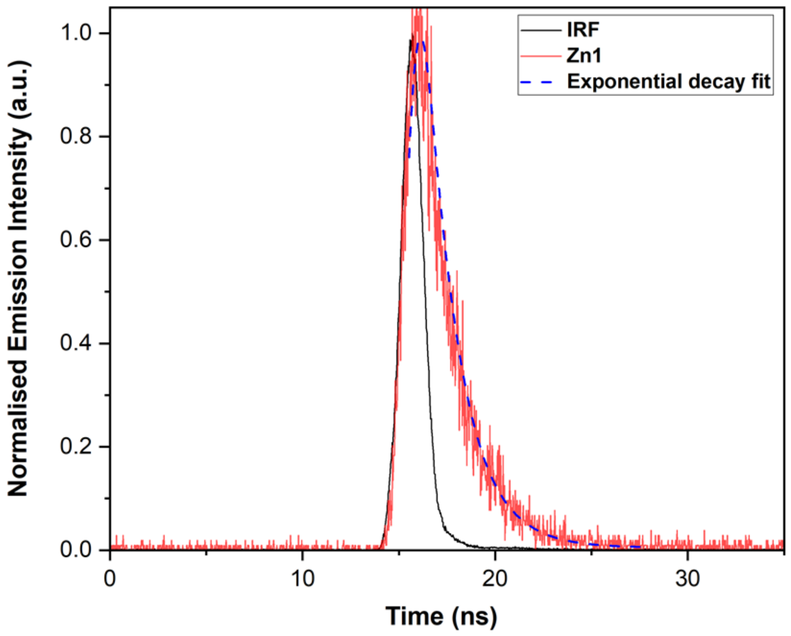

Figure S22. The lifetime of emission for **Zn1** in deoxygenated chloroform at a concentration of 0.5 μM, fitted to a mono-exponential decay with an IRF via reconvolution fitting.

5. Polystyrene PSP performance Studies

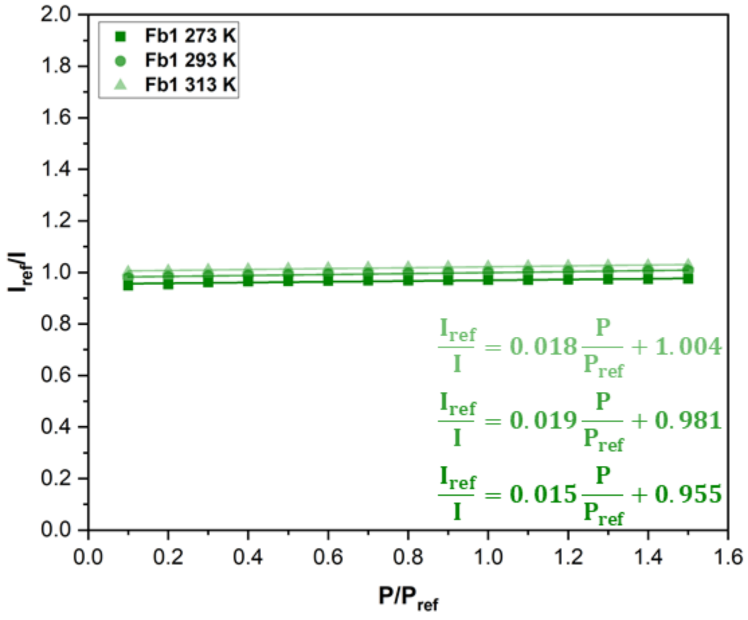

Figure S23. The modified Stern-Volmer calibrated luminescence response to pressure with associated linear fits of **Fb1** polystyrene PSP. I_{ref} and P_{ref} are the luminescence intensity and pressure at 100 kPa and 293 K. Inset shows the structure of **Fb1**.

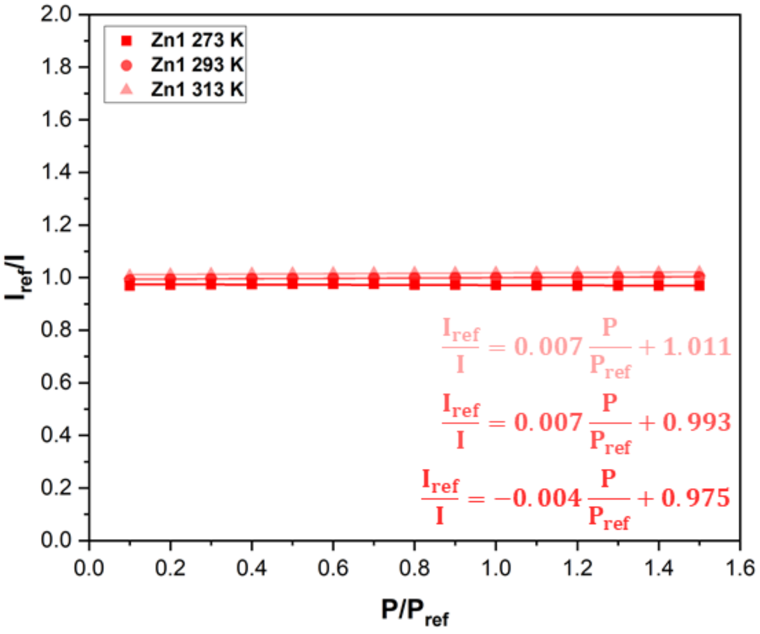

Figure S24. The modified Stern-Volmer calibrated luminescence response to pressure with associated linear fits of **Zn1** polystyrene PSP. I_{ref} and P_{ref} are the luminescence intensity and pressure at 100 kPa and 293 K. Inset shows the structure of **Zn1**.

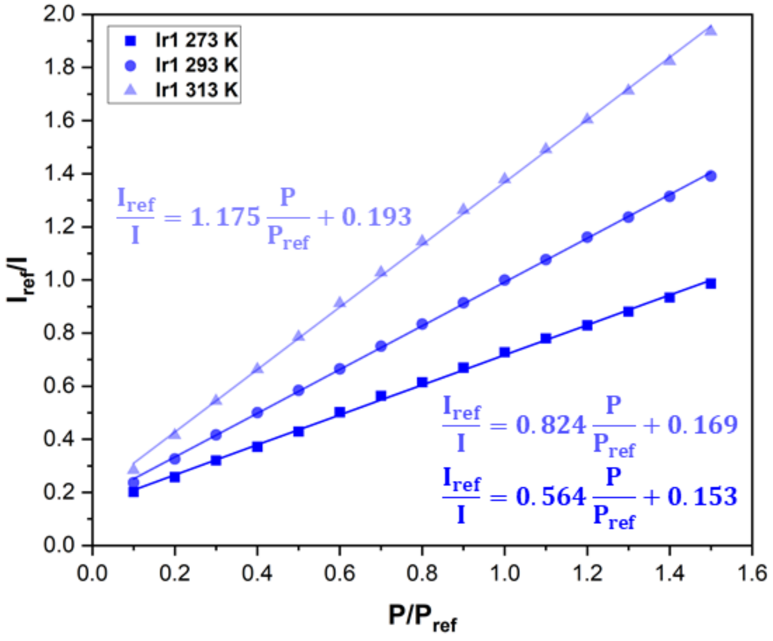

Figure S25. The modified Stern-Volmer calibrated luminescence response to pressure with associated linear fits of **Ir1** polystyrene PSP. I_{ref} and P_{ref} are the luminescence intensity and pressure at 100 kPa and 293 K. Inset shows the structure of **Ir1**.

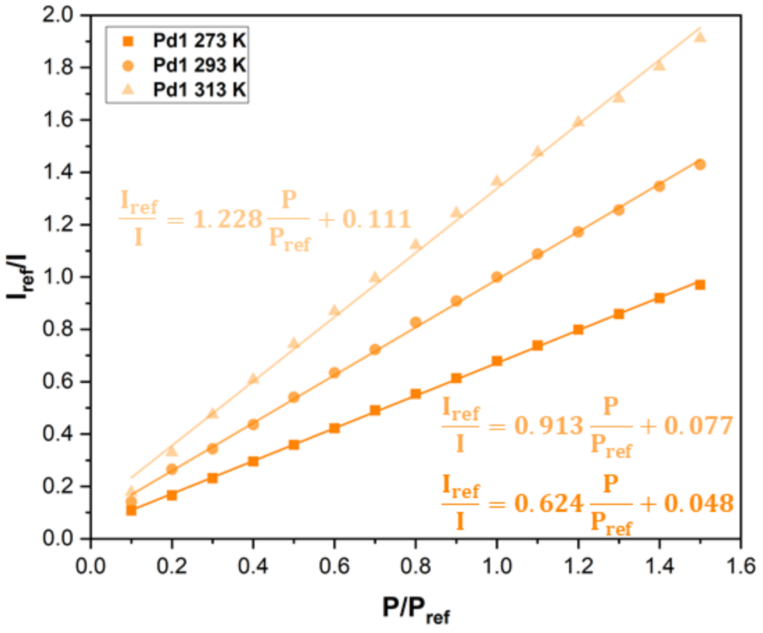

Figure S26. The modified Stern-Volmer calibrated luminescence response to pressure with associated linear fits of **Pd1** polystyrene PSP. I_{ref} and P_{ref} are the luminescence intensity and pressure at 100 kPa and 293 K. Inset shows the structure of **Pd1**.

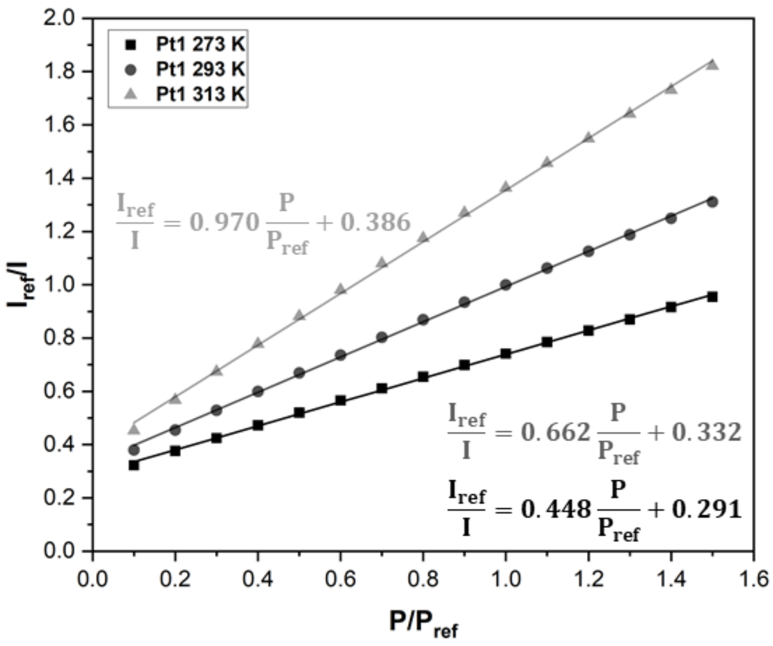

Figure S27. The modified Stern-Volmer calibrated luminescence response to pressure with associated linear fits of **Pt1** polystyrene PSP. I_{ref} and P_{ref} are the luminescent intensity and pressure at 100 kPa and 293 K. Inset shows the structure of **Pt1**.

6. Crystallographic data

Data collection: X-ray diffraction data for compounds **Zn1**, **Ir1** and **Pd1** collected using a dual wavelength Rigaku FR-X rotating anode diffractometer using CuKα (λ = 1.54146 Å) radiation, equipped with an AFC-11 4 circle goniometer, VariMAX^{TM} microfocus optics, a Hypix-6000HE detector and an Oxford Cryosystems 800 plus nitrogen flow gas system, at a temperature of 100K. Data were collected and reduced using CrysAlisPro v42.[2] Absorption correction was performed using empirical methods (SCALE3 ABSPACK) based upon symmetry-equivalent reflections combined with measurements at different azimuthal angles.

Crystal structure determination and refinements: The crystal structure was solved and refined against all F2 values using the SHELX and Olex2 suite of programmes.[3,4] All non-hydrogen atoms were refined anisotropically. Hydrogen atoms were constrained to idealised positions with the coordinates refined to ride with the parent atom. Hydrogen isotropic atomic displacement parameters were constrained to ride with the parent atom with an appropriate multiplier for the hybridisation. In the case of **Ir1**, disordered solvent dichloromethane and chloroform was refined over three positions with two DCM and one chloroform. The occupancies were refined such that the occupancies for the three parts were freely refined against individual free variables but constrained so that the sum of those free variables equals 100% occupied. The dichloromethane and chloroform 1,2- and 1,3- bond distances restrained to be similar.

Crystallographic data have been deposited with the CCDC (CCDC 2376481-2376483).

Table S4. Crystallographic data for **Zn1**, **Ir1** and **Pd1**.

Identification code	**Zn1**	**Ir1**	**Pd1**
Empirical formula	$C_{50}H_{22}F_{20}N_4Zn$	$C_{49}H_{15.35}Cl_{9.65}F_{20}IrN_4O$	$C_{44}H$[illegible]
Formula weight	1124.08	1590.29	107[illegible]
Temperature/K	100.01(10)	100.0(2)	99.9[illegible]
Crystal system	monoclinic	tetragonal	trigo[illegible]
Space group	$P2_1/c$	P4/n	R-3
a/Å	12.60328(12)	16.93580(10)	19.7[illegible]
b/Å	11.61415(12)	16.93580(10)	19.7[illegible]
c/Å	15.06498(17)	9.39590(10)	25.0[illegible]
α/°	90	90	90
β/°	103.9135(10)	90	90
γ/°	90	90	120
Volume/Å^3	2140.46(4)	2694.94(4)	847[illegible]
Z	2	2	9
ρ_{calc}g/cm^3	1.744	1.960	1.90[illegible]
μ/mm^{-1}	2.004	10.240	5.28[illegible]
F(000)	1120.0	1533.0	473[illegible]
Crystal size/mm^3	0.103 × 0.085 × 0.036	0.31 × 0.21 × 0.203	0.35[illegible]
Radiation	Cu Kα (λ = 1.54184)	Cu Kα (λ = 1.54184)	Cu K[illegible]
2Θ range for data collection/°	7.226 to 152.306	7.382 to 151.938	8.74[illegible]
Index ranges	-15 ≤ h ≤ 15, -14 ≤ k ≤ 14, -18 ≤ l ≤ 16	-21 ≤ h ≤ 21, -21 ≤ k ≤ 21, -11 ≤ l ≤ 11	-22 [illegible]
Reflections collected	12613	68591	218[illegible]
Independent reflections	4346 [R_{int} = 0.0215, R_{sigma} = 0.0250]	2818 [R_{int} = 0.0457, R_{sigma} = 0.0113]	380[illegible]
Data/restraints/parameters	4346/0/341	2818/235/259	380[illegible]
Goodness-of-fit on F^2	1.044	1.120	1.07[illegible]
Final R indexes [I>=2σ (I)]	R_1 = 0.0316, wR_2 = 0.0844	R_1 = 0.0330, wR_2 = 0.0849	R_1 = [illegible]
Final R indexes [all data]	R_1 = 0.0358, wR_2 = 0.0869	R_1 = 0.0331, wR_2 = 0.0850	R_1 = [illegible]
Largest diff. peak/hole / e Å^{-3}	0.34/-0.82	1.52/-2.53	0.36[illegible]

7. References

1. Nunn, E. J.; Tsioumanis, D.; Whitehead, G. F. S.; Fisher, T. B.; Roberts, D. A.; Quinn, M. K.; Natrajan, L. S. Exploring the Effect of Porphyrin Chemical Structure on the Performance of Polymer-Based Pressure-Sensitive Paints. *Sens. Actuators B Chem.* **2024**, *409*, 135577. https://doi.org/10.1016/j.snb.2024.135577.

2. Rigaku Oxford Diffraction, (2024), CrysAlisPro Software system, version 1.171.43.116a, Rigaku Corporation, Wroclaw, Poland.

3. Sheldrick, G. M. Crystal Structure Refinement with SHELXL. *Acta Crystallogr. Sect. C Struct. Chem.* **2015**, *71* (1), 3–8. https://doi.org/10.1107/S2053229614024218.
